# Flavone C-Glycosides from *Dianthus superbus* L. Attenuate Metabolic Dysfunction-Associated Steatotic Liver Disease (MASLD) via Multi-Pathway Regulations

**DOI:** 10.3390/nu17152456

**Published:** 2025-07-28

**Authors:** Ming Chu, Yingying Tong, Lei Zhang, Yu Zhang, Jun Dang, Gang Li

**Affiliations:** 1 Center for Mitochondria and Healthy Aging, College of Life Sciences, Yantai University, Yantai 264005, China; 18136875242@163.com (M.C.); zhanglei982024@163.com (L.Z.); zhangyu_ytu@126.com (Y.Z.); 2 Qinghai Provincial Key Laboratory of Tibetan Medicine Research, Northwest Institute of Plateau Biology, Chinese Academy of Sciences, Xining 810001, China; 15371590989@163.com; 3 Jiangsu Collaborative Innovation Center of Chinese Medicinal Resources Industrialization, Nanjing University of Chinese Medicine, 138 Xianlin Road, Nanjing 210023, China

**Keywords:** MASLD, *Dianthus superbus* L., flavone C-glycosides, lipid metabolism, insulin resistance, inflammation, apoptosis

## Abstract

Background: The metabolic dysfunction-associated steatotic liver disease (MASLD) represents an escalating global health concern, with effective treatments still lacking. Given its complex pathogenesis, multi-targeted strategies are highly desirable. Methods: This study reports the isolation of four flavone C-glycosides (FCGs) from *Dianthus superbus* L. and explores their potential in treating MASLD. The bioactivity and underlying mechanisms of FCGs were systematically evaluated by integrating network pharmacology, molecular docking, and zebrafish model validation. Results: Network pharmacology analysis revealed that FCGs may modulate multiple MASLD-related pathways, including lipid metabolism, insulin signaling, inflammation, and apoptosis. Molecular docking further confirmed strong binding affinities between FCGs and key protein targets involved in these pathways. In the zebrafish model of MASLD induced by egg yolk powder, FCGs administration markedly attenuated obesity, hepatic lipid accumulation, and liver tissue damage. Furthermore, FCGs improved lipid metabolism and restored locomotor function. Molecular analyses confirmed that FCGs upregulated PPARγ expression to promote lipid metabolism, restored insulin signaling by enhancing INSR, PI3K, and AKT expression, and suppressed inflammation by downregulating TNF, IL-6 and NF-κB. Additionally, FCGs inhibited hepatocyte apoptosis by elevating the BCL-2/BAX ratio. Conclusions: These findings highlight the multi-pathway regulatory effects of FCGs in MASLD, underscoring its potential as a novel therapeutic candidate for further preclinical development.

## 1. Introduction

Metabolic dysfunction-associated steatotic liver disease (MASLD), formerly known as non-alcoholic fatty liver disease (NAFLD), comprises a spectrum of hepatic pathologies defined by ectopic lipid deposition stemming from metabolic dysregulation. Afflicting approximately 38% of the global population and continuing to rise in prevalence [1,2], this pervasive chronic condition represents an urgent public health priority demanding accelerated investigation into its pathogenesis and novel therapeutics.

The pathogenesis of MASLD is multifactorial, involving insulin resistance, dysregulated lipid metabolism, oxidative stress, inflammatory responses, gut microbiota imbalance, and genetic factors [3]. Among these, the progression of MASLD is significantly influenced by peroxisome proliferator-activated receptor γ (PPARγ), a key lipid metabolism regulator [4]. The PPARγ plays a central role in regulating fatty acid storage, metabolism, and oxidation, maintaining lipid homeostasis. When PPARγ activity is disrupted, fatty acid oxidation is impaired, leading to excessive hepatic lipid accumulation, insulin resistance, and inflammation, key drivers of MASLD progression [5]. Additionally, Insulin resistance is closely associated with dysfunction in the insulin signaling cascade. Under physiological conditions, insulin binding to the insulin receptor (INSR), the phosphoinositide 3-kinase (PI3K)-protein kinase B (AKT) pathway is activated, essential for maintaining metabolic balance in the liver [6]. However, impairment of this pathway, however, leads to increased hepatic fatty acid uptake and lipid deposition, further aggravating MASLD [7]. Inflammatory is another critical factor in disease progression. Transcription factors such as nuclear factor kappa B p65 subunit (RELA) and p50 subunit (NF-κB1) promote chronic liver inflammation and fibrosis by activating pro-inflammatory cytokines, including tumor necrosis factor (TNF) and interleukin-6 (IL-6) [8,9].

Concurrently, fatty acid accumulation-induced cellular stress and inflammation exacerbate hepatocyte injury through apoptosis. An imbalance mediates this process between B-cell lymphoma-2 (BCL-2) and BCL-2-associated X protein (BAX), ultimately contributing to liver injury [10]. These interrelated mechanisms of metabolic dysregulation, insulin resistance, inflammation, and apoptosis drive the initiation and progression of MASLD through a complex network of signaling pathways.

Current MASLD management primarily relies on non-pharmacological interventions, such as weight loss and lifestyle modifications [11]. However, these approaches often yield limited efficacy and are challenging to sustain long-term. Pharmacological options remain scarce, particularly in early-stage MASLD, with most treatments lacking robust validation. In 2024, Resmetirom became the first FDA-approved drug for MASLD [12]. Although clinical trials have confirmed its therapeutic benefits, its single-target mode of action restricts its effectiveness in addressing the complex, multifactorial nature of MASLD and the heterogeneity among patients [13,14]. Consequently, the development of multi-targeted drugs capable of addressing the complex pathological processes of MASLD remains an urgent unmet need.

*Dianthus superbus* L., a traditional herbal medicine predominantly found in high-altitude regions, belongs to the Caryophyllaceae family. Traditional herbal medicines and their natural products have garnered increasing attention as potential therapeutic agents for MASLD [15,16]. Although *Dianthus superbus* L. and its components’ effects on MASLD have not been fully studied, the plant’s hepatoprotective, anti-inflammatory, antioxidant, and anti-diabetic nephropathy qualities are well-established, indicating significant thera-peutic promise [17,18,19,20,21]. Flavonoids, the primary bioactive components of *Dianthus superbus* L., have emerged as a research hotspot due to their diverse biological activities [22,23]. Studies indicate that flavonoids positively modulate key pathological processes in MASLD, including lipid metabolism regulation, insulin resistance improvement, inflammation inhibition, and oxidative stress alleviation [24,25]. These multi-target effects are well-suited to address the complex and interconnected mechanisms underlying MASLD, underscoring the promise of flavonoids as potential therapeutic agents.

In this study, we isolated four flavone C-glycosides (FCGs) from *Dianthus superbus* L., and potential therapeutic targets for MASLD were predicted using network pharmacology and molecular docking. Using a zebrafish model, we evaluated the efficacy of FCGs in alleviating MASLD and further elucidated their underlying mechanisms. Our findings not only provide a theoretical foundation for developing novel therapeutic strategies for MASLD but also underscore the potential of *Dianthus superbus* L. and its flavone C-glycosides as promising candidates for further research and application.

## 2. Materials and Methods

### 2.1. Reagents

Egg yolk powder was purchased from Shanghai Yuanye Bio-Technology Co., Ltd. (Shanghai, China). Larval AP100 was obtained from Zeigler Bros., Inc. (Gardners, PA, USA). Assay kits for triglyceride (TG), total cholesterol (TC), low-density lipoprotein cholesterol (LDL-C), and high-density lipoprotein cholesterol (HDL-C) were acquired from Nanjing Jiancheng Bioengineering Institute (Nanjing, China). Oil Red O and tricaine powder were sourced from Sigma-Aldrich (St. Louis, MO, USA). Hematoxylin and eosin stains were supplied by Zhongshan Golden Bridge Biotechnology Co., Ltd. (Beijing, China). Fenofibrate was purchased from Aladdin Reagent Co., Ltd. (Shanghai, China). The Enhanced BCA Protein Assay Kit was provided by Biodee Biotechnology Co., Ltd. (Beijing, China). The SPARKeasy Improved Tissue/Cell RNA Kit, SPARKscript II RT Plus Kit, and 2 × SYBR Green qPCR Mix were obtained from Shandong Sikejie Biotechnology Co., Ltd. (Jinan, China). Primers were synthesized by Sangon Biotech Co., Ltd. (Shanghai, China).

### 2.2. Plant Sample Origin

*Dianthus superbus* L. was collected from the Laoshan District of Qingdao, Shandong Province, China, at an elevation of 1132.7 m (36°10′ N, 120°37′ E). The specimen was authenticated by Prof. Jiantao Lv (College of Pharmacy, Yantai University) and is deposited at Yantai University.

### 2.3. Zebrafish Maintenance

AB strain zebrafish (wild-type; supplier: Hubei Chuangxin Biotechnology, Wuhan, China) were maintained in an Aisheng recirculating system (Beijing, China). Feeding protocols included twice-daily provision of live Artemia nauplii. The temperature was maintained at 28 ± 0.5 °C, with a conductivity of 450–500 μS/cm, pH 7.0–8.0, and a 14 h/10 h light/dark cycle. For mating, zebrafish were transferred to mating boxes at a male-to-female ratio of 3:2 in the evening and separated by a divider. The divider was removed the following morning to allow mating and egg collection. Fertilized eggs were collected approximately 2 h later, transferred to E3 medium, and cultured for subsequent experiments.

### 2.4. Isolation and Purity of Sample

In the first-dimensional separation (7-X10 preparative column, 20 × 250 mm, 7 µm), fraction 1 (2.1 g) and fraction 2 (0.6 g) were previously obtained from *Dianthus superbus* L. (12.2 g) using medium-pressure preparative chromatography and high-pressure liquid chromatography [26]. Fraction 1 was further separated using a Sunfire^®^ C18 column (Waters, Milford, MA, USA), while fraction 2 was separated using a Click XION column (Acchrom Technologies, Beijing, China), as detailed in the Supporting Information (Experimental section). The isolated compounds were verified for purity and structural characterization.

### 2.5. Network Pharmacology Analysis

#### 2.5.1. Identification of Compound-Disease Intersection Targets

The chemical structures of the isolated FCGs were converted into SMILES format. Potential targets of these compounds were predicted using the Swiss Target Prediction and SEA Search Server platforms. MASLD-related disease targets were retrieved from the GeneCards, OMIM, and TTD databases. The target data were standardized using UniProt. The intersection between predicted compound targets and MASLD-associated targets was then identified using Venny 2.1.0 to identify potential FCG targets for MASLD intervention.

#### 2.5.2. PPI Network and Core Target Analysis

To establish a protein–protein interaction (PPI) network, the discovered targets were entered into the STRING 12.0 database. After that, the generated network was loaded into Cytoscape 3.10.1 for further analysis and visualization. By assessing important topological parameters, such as degree centrality, betweenness centrality, and proximity centrality, core targets were found.

#### 2.5.3. GO and KEGG Enrichment Analysis of Potential Targets

The intersection targets were analyzed using the Metascape platform. The GO functional annotation and KEGG pathway enrichment analyses were performed, and the results were visualized.

#### 2.5.4. Construction of the Compound-Target-Pathway Network

The compound-target-pathway network was constructed by importing potential targets and enriched pathways into Cytoscape 3.10.1 software. The network was analyzed using the CytoNCA plugin, and nodes were color-coded based on their degree values.

#### 2.5.5. Molecular Docking Analyses

The 2D structures of the compounds were converted to 3D using Chem3D 16.0. Protein crystal structures, including AKT1, RELA, NF-κB1, TNF, IL-6, BCL-2, PPARγ, PI3K, and BAX, were downloaded from the RCSB Protein Data Bank. Molecular docking and binding energy analyses using AutoDock followed PyMOL structural preprocessing. A lager negative binding energy value indicates greater conformational stability and a stronger ligand–protein interaction. It is generally accepted that −5.0 kcal/mol serves as the threshold binding energy for determining the stability of ligand–receptor complexes [27].

### 2.6. Animal Experiments

#### 2.6.1. Zebrafish Treatment

Healthy 5 days post-fertilization (dpf) zebrafish larvae were randomly assigned to groups. The control group was fed standard AP100 feed, while the model group, positive control group, and FCGs groups were exposed to 0.2% egg yolk powder [28]. Based on preliminary experiments, the positive control group was administered 1.5 μM Fenofibrate (Fnf), whereas the FCG groups received 10 μM of FCG1, FCG2, FCG3, or FCG4, respectively. The culture medium was changed daily, and feeding was performed once per day for five consecutive days.

#### 2.6.2. Weight, Length, and BMI Measurements

After a 24-h fast, zebrafish were anesthetized with 0.016% tricaine for 3 min. Body length (from the mouth to the base of the tail fin) was measured under an inverted microscope (DMi1, Leica, Wetzlar, Germany) using CellSens Entry software 4.2. For weight measurement, excess water was removed from the fish’s surface using filter paper before weighing on an analytical balance. Body Mass Index (BMI) was calculated as weight/(length^2^).

#### 2.6.3. Lipid Metabolism Biochemical Assays

Thirty zebrafish were homogenized in 9 volumes of pre-chilled PBS (g/mL) for each group, and centrifuged at 2500 rpm for 15 min to collect the supernatant. The total protein content was determined using a BCA protein assay kit. Test kits were used to evaluate lipid metabolic parameters such as TG, TC, LDL-C, and HDL-C as directed.

#### 2.6.4. Oil Red O Staining of Whole Zebrafish

After fasting, zebrafish were anesthetized, washed with PBS, and fixed overnight in 4% paraformaldehyde at 4 °C. Following PBS washing, the fish were dehydrated in 60% isopropanol for 30 min and then incubated in freshly prepared 0.3% Oil Red O solution in the dark for 3 h. After incubation, the fish were washed and decolorized in 60% isopropanol and PBS until the signal was clear [29]. Hepatic lipid accumulation was visualized under a microscope, and representative images were captured. Staining intensity was quantified using ImageJ software 1.52.

#### 2.6.5. Liver Tissue Sectioning and Hematoxylin and Eosin (HE) Staining

After overnight fixation in 4% paraformaldehyde, zebrafish were dehydrated, embedded in paraffin, and sectioned at 5 μm thickness. Deparaffinized and rehydrated sections were hematoxylin-stained (8 min), rinsed in water, and differentiated in 1% hydrochloric acid alcohol (~30 s) before another water wash. Subsequent eosin staining (5 min) was followed by ethanol gradient dehydration, xylene clearing, and neutral resin mounting. Microscopic analysis and image collection were performed [30].

#### 2.6.6. Behavioral Analysis

After drug treatments, zebrafish were transferred to a 48-well plate (one fish per well) containing 500 μL of E3 medium. Fish movement was recorded using a zebrafish behavioral tracking system (DanioVision, Noldus, Beijing, China) and analyzed using EthoVision XT software (SMART 3.0). The experiment was conducted under white light at a constant temperature of 28 °C. After a 10-min acclimation period, fish movement was recorded and analyzed for 10 min [31].

#### 2.6.7. Real-Time Quantitative PCR (qPCR) Assays

Total RNA was isolated from 30 zebrafish per group using the Tissue/Cell RNA Kit. The concentration and purity of RNA were evaluated using a micro-volume spectrophotometer (ND5000, BioTeke, Wuxi, China). According to the SPARKscript II RT Plus Kit instructions, 1 μg of total RNA was used to synthesize cDNA. The qPCR reaction mixture was prepared with 2 × SYBR Green qPCR Mix, and amplification was performed on a qPCR System (7500 Fast, Thermo Fisher Scientific, Waltham, MA, USA) under cycling conditions: 95 °C, 3 min; 40 cycles of 95 °C (15 s), 60 °C (20 s), 72 °C (30 s). Relative quantification employed β-actin as the endogenous control. Primer sequences are listed in Table 1.

### 2.7. Statistical Analysis

All experiments were performed in triplicate, and data are presented as mean ± standard deviation (Mean ± SD). Statistical analyses were conducted using GraphPad Prism 8.3.0. T-tests were used to determine statistical significance, with *p* < 0.05 considered statistically significant and *p* < 0.01 considered highly significant.

## 3. Results

### 3.1. Preparative Isolation, Purity Analysis and Structural Characterization of Compounds

**Figure 1:**
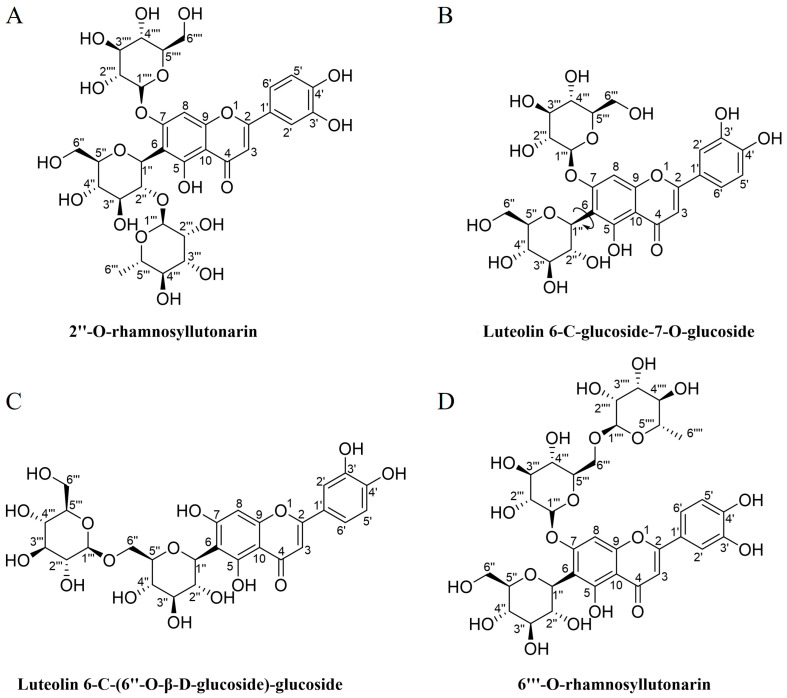
Chemical structural formulas of four flavone C-glycosides (FCGs) from *Dianthus superbus* L. (**A**) 2″-O-rhamnosyllutonarin. (**B**) Luteolin 6-C-glucoside-7-O-glucoside. (**C**) Luteolin 6-C-(6″-O-β-D-glucoside)-glucoside. (**D**) 6‴-O-rhamnosyllutonarin.

Four FCGs were isolated with purity of over 95% and identified from *Dianthus superbus* L.: 2″-O-rhamnosyllutonarin (FCG 1) [32,33], luteolin 6-C-glucoside-7-O-glucoside (FCG 2) [32], luteolin 6-C-(6″-O-β-D-glucoside)-glucoside (FCG 3) [34], and 6‴-O-rhamnosyllutonarin (FCG 4) [35], as detailed in the Supporting Information (Figure S1). Notably, FCG 1 is a newly discovered compound. The chemical structures of these flavone C-glycosides are illustrated in Figure 1.

### 3.2. Network Pharmacology-Based Analysis of Potential Targets and Pathways for FCGs in the Treatment of MASLD

**Figure 2:**
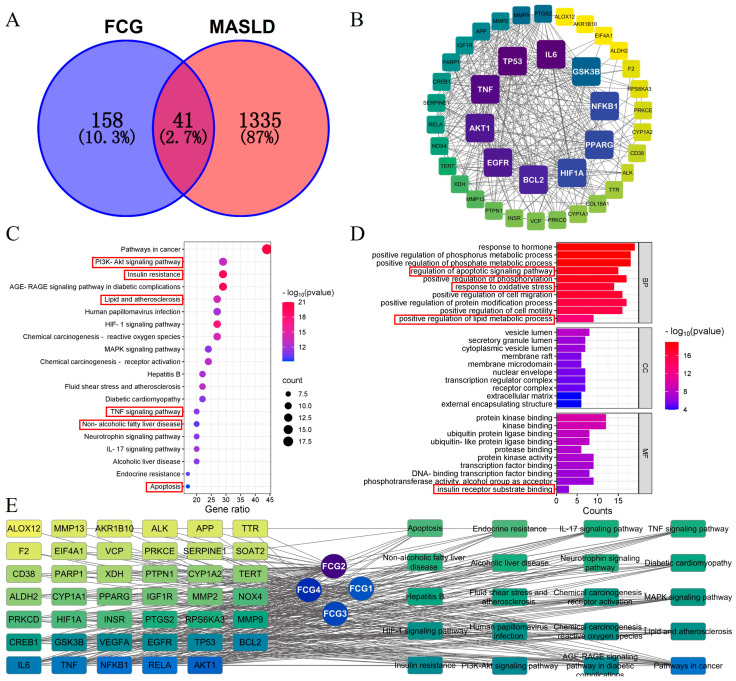
Network pharmacology-based analysis of potential targets and pathways of FCGs in the treatment of metabolic dysfunction-associated steatotic liver disease (MASLD). (**A**) Venny diagram showing the overlap of FCGs and MASLD targets. (**B**) Protein-protein interaction (PPI) network of potential action targets of FCGs in MASLD. (**C**) GO function enrichment analysis of potential action targets. (**D**) KEGG pathway enrichment analysis of potential action targets. (**E**) Network of FCG-Targets-Pathways.

A total of 199 predicted targets for FCGs and 1376 MASLD-related disease targets were intersected, yielding 41 potential targets for FCGs interventions in MASLD (Figure 2A). A PPI network was constructed for these potential targets, followed by network topology analysis. The top ten core targets were identified, as shown in Figure 2B, where the intensity of node colors reflects the strength of interactions. The potential targets mostly involve biological processes and signaling pathways associated with insulin resistance, lipid metabolism, inflammation, and apoptosis, according to the GO and KEGG enrichment analyses (Figure 2C,D). Furthermore, an FCGs-target-pathway network was constructed and analyzed, with results presented in Figure 2E. Network pharmacology analysis preliminarily suggests that FCGs have potential therapeutic effects on MASLD, with FCG2 emerging as the most promising component for improving MASLD. Key targets, including AKT1, RELA, NF-κB1, TNF, IL-6, BCL-2, and PPARγ, may play critical roles, primarily through mechanisms involving insulin resistance, lipid metabolism, inflammation, and apoptosis.

### 3.3. Molecular Docking Analysis of FCGs with Core Therapeutic Targets

**Figure 3:**
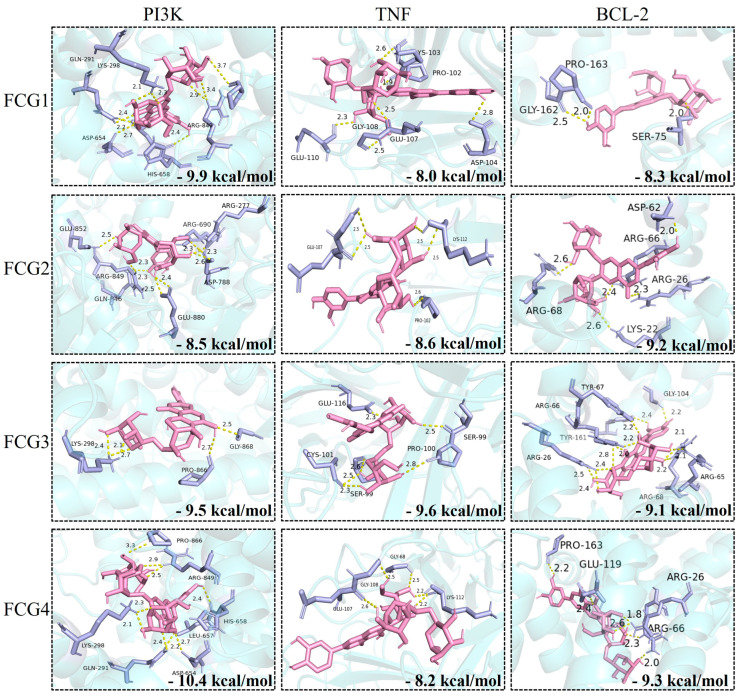
Binding conformations and stability of FCGs with PI3K, TNF, and BCL-2.

To further validate the binding potential of compounds with core targets at the structural level, molecular docking studies were performed on key targets identified through network pharmacology, including AKT1, RELA, NF-κB1, TNF, IL-6, BCL-2, and PPARγ, as well as PI3K and BAX, which play crucial roles in the associated signaling pathways. The binding energies and key binding residues of FCGs with each target protein are summarized in Table 2. Docking results revealed that all FCGs exhibited binding energies below −5.0 kcal/mol across the selected core targets, indicating favorable binding affinity. Notably, FCGs showed particularly strong interactions with PI3K, TNF, and BCL-2, with binding energies below −8.0 kcal/mol, and the detailed interaction modes are illustrated in Figure 3. Among the tested compounds, FCG4 exhibited the strongest binding to PI3K, with a binding energy of −10.4 kcal/mol, suggesting a high potential for biological activity through this interaction.

### 3.4. Effect of FCGs on Obesity in MASLD Zebrafish

**Figure 4:**
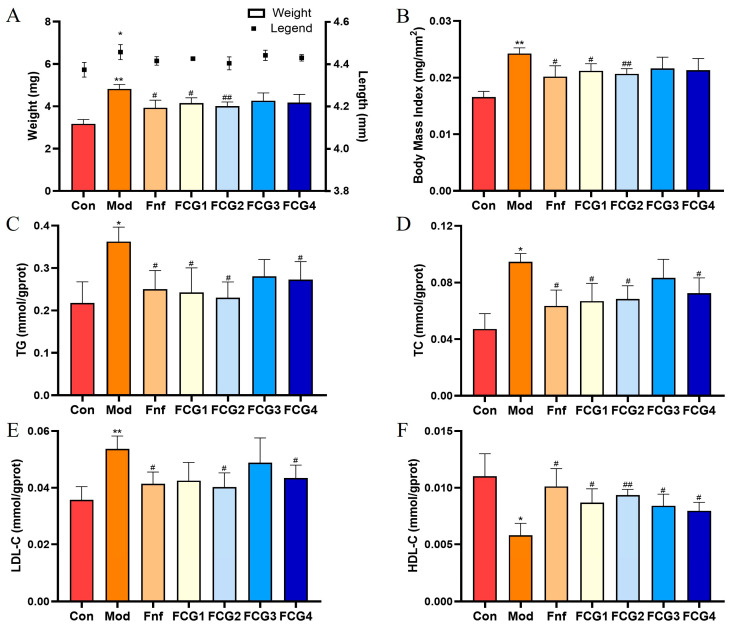
FCGs improved obesity and lipid metabolism disorders in MASLD zebrafish. (**A**) Average weight and average length of zebrafish. (**B**) BMI of zebrafish. (**C**) TG, (**D**) TC, (**E**) LDL-C, and (**F**) HDL-C statistical analysis results. Con: AP100; Mod: 0.2% egg yolk powder; Fnf: 0.2% egg yolk powder + 1.5 μM Fnf; FCG1: 0.2% egg yolk powder + 10 μM FCG1; FCG2: 0.2% egg yolk powder + 10 μM FCG2; FCG3: 0.2% egg yolk powder + 10 μM FCG3; FCG4: 0.2% egg yolk powder + 10 μM FCG4. * *p* < 0.05, ** *p* < 0.01 vs. the control group; # *p* < 0.05, ## *p* < 0.01 vs. the Model group.

To validate the results of the network pharmacology analysis, we successfully established a MASLD zebrafish model by feeding the fish a 0.2% egg yolk powder diet. Given the strong association between obesity and MASLD, we evaluated the effects of egg yolk powder feeding and compound interventions on obesity by measuring the length, weight, and body mass index (BMI) of zebrafish larvae. As shown in Figure 4A,B, zebrafish exposed to egg yolk powder for five consecutive days showed significant length, weight, and BMI increases compared to the control group (*p* < 0.05, *p* < 0.01). Following intervention with FCGs and the positive control (Fnf), improvements in obesity-related parameters were observed. FCG1, FCG2, and Fnf significantly reduced body weight and BMI relative to the model group (*p* < 0.05, *p* < 0.01), demonstrating their efficacy in mitigating high-fat diet-induced obesity.

### 3.5. Effect of FCGs on Lipid Metabolism in MASLD Zebrafish

The results of lipid metabolism marker assays are shown in Figure 4C–F. Zebrafish in the MASLD model group had significantly higher levels of TG, TC, and LDL-C (*p* < 0.05, *p* < 0.01) and lower levels of HDL-C (*p* < 0.05) than the control group on a normal diet. Treatment with the positive control, Fnf, significantly improved lipid metabolism disorders in MASLD zebrafish (*p* < 0.05). Similarly, FCGs interventions effectively alleviated these metabolic disturbances, with FCG2 and FCG4 showing significant improvements across all lipid parameters (*p* < 0.05, *p* < 0.01).

### 3.6. Effect of FCGs on Hepatic Lipid Accumulation in MASLD Zebrafish

**Figure 5:**
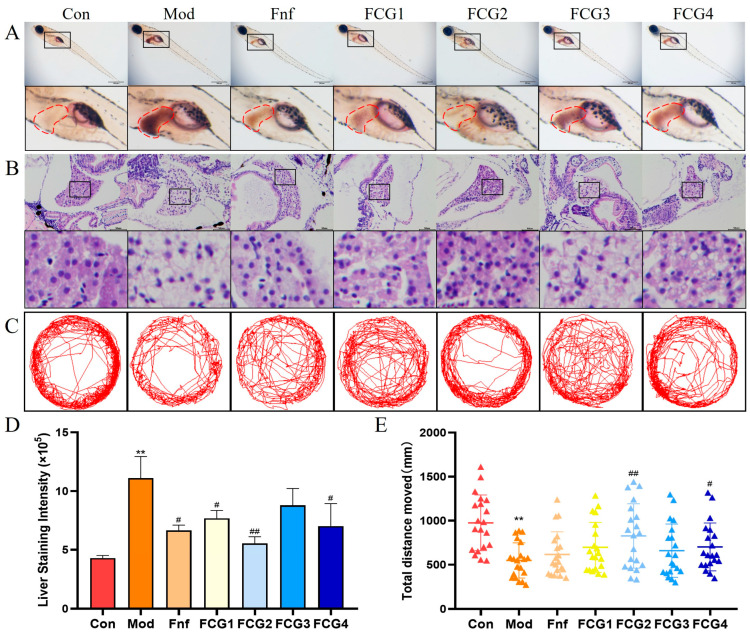
FCGs alleviated hepatic lipid accumulation and tissue damage while improving locomotor activity in MASLD zebrafish. (**A**) Whole fish Oil Red O staining results (40×, the red area indicates the liver). (**B**) HE staining results of liver tissue sections (400×). (**C**) Zebrafish movement trajectory. (**D**) Quantitative analysis of Oil Red O staining in the liver region of zebrafish. (**E**) Statistical analysis of total movement distance in zebrafish. ** *p* < 0.01 vs. the control group; # *p* < 0.05, ## *p* < 0.01 vs. the Mod.

As shown in Figure 5A, the liver region of zebrafish in the control group showed minimal staining, indicating low lipid content. However, the model group exhibited intense staining, reflecting substantial lipid accumulation. In contrast, the Fnf positive control group showed a marked reduction in hepatic lipid droplets. Notably, FCG1, FCG2, and FCG4 showed markedly lighter hepatic staining than the model group, comparable to the positive control, indicating potent inhibition of lipid accumulation. Quantitative analysis (Figure 5D) confirmed significantly higher staining intensity in the model group versus controls (*p* < 0.01). All treatment groups (Fnf, FCG1, FCG2, FCG4) exhibited reduced staining versus model (*p* < 0.05 or *p* < 0.01). And the quantitative value of FCG2 group was close to that of the control group, indicating that lipid accumulation in the livers of juvenile fish had returned to near-normal levels.

### 3.7. Effect of FCGs on Hepatic Tissue Injury in MASLD Zebrafish

Further histological examination using HE staining (Figure 5B) revealed distinct differences in liver tissue morphology across groups. The control group exhibited normal liver structure, characterized by orderly cell arrangement, uniformly stained nuclei, and intact cytoplasm. The model group displayed significant ballooning degeneration, with enlarged hepatocytes, loose and transparent cytoplasm, and nuclear vacuolation, all of which are indicators of fatty degeneration and liver damage. Following Fnf and FCGs interventions, liver tissue morphology showed improvement. Although some vacuolation persisted, the overall structure became denser, and pathological changes were markedly reduced, suggesting a trend toward normalization. These findings align with the Oil Red O staining results, further supporting the beneficial effects of FCGs in ameliorating MASLD.

### 3.8. Effect of FCGs on Behavioral Indicators of MASLD Zebrafish

To further evaluate the effects of FCGs on MASLD zebrafish, we assessed locomotor activity across different groups. Analysis of movement trajectories revealed that the control group exhibited higher activity levels, while the model group showed a sparser movement pattern, indicating significantly reduced locomotor activity. Zebrafish treated with Fnf and FCGs demonstrated improved locomotor activity compared to the model group (Figure 5C). These observations were validated by quantitatively examining the overall movement distance (Figure 5E): Compared to the control group, the model group’s movement distances were significantly lower (*p* < 0.01). The Fnf group, in comparison, displayed a moderate enhancement. All FCG-treated groups showed increased locomotor activity; however, the most significant gains (*p* < 0.01) were produced by FCG2 and FCG4, indicating that they may be able to address the motor deficits associated with MASLD.

### 3.9. Effect of FCGs on mRNA Expression Levels in MASLD Zebrafish

**Figure 6:**
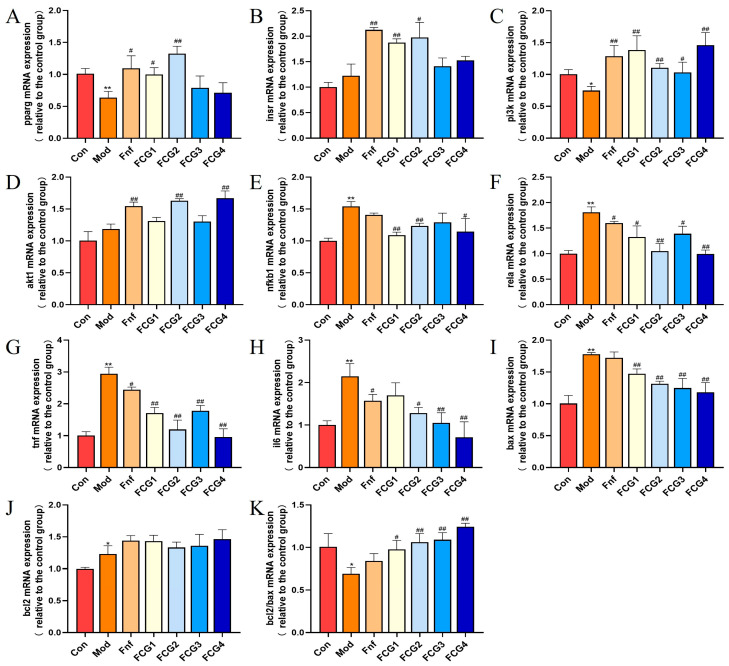
FCGs improved abnormal mRNA expression levels in MASLD zebrafish. (**A**) Expression levels of *pparg*, (**B**) *insr*, (**C**) *pi3k*, (**D**) *akt1*, (**E**) *nfkb1*, (**F**) *rela*, (**G**) *tnf*, (**H**) *il6*, (**I**) *bax*, (**J**) *bcl2*, and (**K**) *bcl2*/*bax* mRNA expression levels. * *p* < 0.05, ** *p* < 0.01 vs. the control group; # *p* < 0.05, ## *p* < 0.01 vs. the Model group.

In preliminary experiments, FCGs exhibited significant anti-MASLD activity. To further explore their mechanisms of action, we evaluated the expression of key targets predicted by network pharmacology in different treatment groups. The mRNA expression of *pparg* was significantly downregulated in MASLD zebrafish (*p* < 0.01), while FCGs treatment significantly reversed this effect, upregulating *pparg* expression (*p* < 0.05, *p* < 0.01) (Figure 6A). Additionally, genes associated with the insulin signaling pathway were dysregulated in the model group. FCGs upregulated the expression of *insr*, *pi3k*, and *akt1*, thereby restoring insulin signal transduction. Notably, all these improvements in the FCG2 group reached statistical significance (*p* < 0.05, *p* < 0.01) (Figure 6B–D). In MASLD zebrafish, the expression of inflammation-related genes (*nfkb1*, *rela*, *tnf*, and *il6*) was significantly elevated (*p* < 0.01). These pro-inflammatory markers were significantly downregulated after receiving FCG treatment; the majority of these changes were statistically significant (*p* < 0.05, *p* < 0.01), suggesting a potent anti-inflammatory effect (Figure 6E–H). In the model group, the expression levels of *bcl2* and *bax* were significantly increased (*p* < 0.05, *p* < 0.01), accompanied by a significant decrease in the *bcl2*/*bax* ratio (*p* < 0.05). Compared to the model group, FCGs treatments significantly increased the *bcl2*/*bax* ratio (*p* < 0.05, *p* < 0.01), suggesting a reduction in apoptosis (Figure 6I–K).

## 4. Discussion

The MASLD is a globally prevalent chronic liver disorder. Unhealthy dietary habits and sedentary lifestyles have led to a rising global incidence of MASLD, with an increasing proportion of patients progressing to advanced stages [36]. Despite its rising burden, effective therapeutic options remain scarce, highlighting the urgent need for novel treatment strategies.

The traditional herbal remedy *Dianthus superbus* L. is well-known for its anti-inflammatory, antioxidant, hepatoprotective, and anti-diabetic nephropathy properties [37]. Building on previous studies from our laboratory, which revealed its anti-inflammatory activity and potential to ameliorate glucose metabolism disorders [18,26], this study successfully isolated four FCGs using a two-dimensional high-performance liquid chromatography (2D-HPLC) system. Compared to conventional flavonoid O-glycosides, FCGs exhibit superior structural stability and bioavailability, enhancing their therapeutic potential [38].

Network pharmacology analysis indicated that FCGs may ameliorate MASLD through multi-target mechanisms, including modulation of insulin resistance, lipid metabolism, inflammation, and apoptosis. This aligns with the well-documented broad-spectrum activity of flavonoids, reinforcing their potential utility in MASLD treatment [24]. To further substantiate these findings, we employed molecular docking to validate, at the structural level, the binding potential of FCGs with key targets predicted by network pharmacology, including AKT1, RELA, NF-κB1, TNF, IL-6, BCL-2, and PPARγ, as well as PI3K and BAX, which play pivotal roles in the associated signaling pathways. All FCGs exhibited binding energies lower than −5.0 with the target proteins, particularly showing values below −8.0 when bound to PI3K, TNF, and BCL-2. Notably, FCG4 demonstrated a strong binding affinity toward PI3K, with a binding energy of −10.4, indicating a highly stable interaction.

Zebrafish, a vertebrate model with high genetic and metabolic homology to mammals, offers advantages such as rapid reproduction, ex vivo development, and optical transparency, making it ideal for liver and metabolic research [39]. Its lipid metabolism closely mirrors human pathophysiology [40,41]. In this study, a MASLD zebrafish model was established via a 0.2% egg yolk powder diet. Treatment with FCGs significantly mitigated MASLD-related phenotypes, including obesity, dyslipidemia, hepatic lipid accumulation, and liver tissue damage. Notably, FCGs-treated zebrafish exhibited restored locomotor activity, as evidenced by increased swimming distance—a finding consistent with behavioral improvements reported in hyperlipidemia models treated with other natural compounds [31]. Among the tested compounds, FCG2 and FCG4 demonstrated superior efficacy. Collectively, these findings highlight FCGs’ therapeutic potential against MASLD. To further elucidate their mechanisms of action, we next examined the mRNA expression levels of key targets using a qPCR assay.

PPARγ, a central regulator of lipid metabolism, inflammation, and insulin sensi-tivity, is a well-recognized therapeutic target in MASLD management [42]. FCGs significantly upregulated *pparg* expression, suggesting their role as potential PPARγ agonists. This activation likely contributes to improved insulin sensitivity and lipid homeostasis, aligning with reported mechanisms of PPARγ-targeted therapies [43].

Insulin resistance, a hallmark of MASLD, disrupts the INSR-PI3K-AKT pathway, exacerbating hepatic lipid accumulation. Under physiological homeostasis, the binding of insulin to the INSR activates the PI3K-AKT signaling pathway, triggering a downstream signal transduction cascade that mediates glucose uptake and maintains metabolic homeostasis [44,45,46]. FCGs upregulated the expression of *insr*, *pi3k*, and *akt1* to varying degrees. This restoration of insulin signaling may reduce hepatic lipogenesis and enhance fatty acid oxidation, counteracting lipid overload [47,48,49].

NF-κB is a key transcription factor in the regulation of immune and inflammatory responses. Upon activation, NF-κB induces the expression of pro-inflammatory cytokines such as TNF and IL-6, initiating inflammatory and fibrotic processes central to MASLD pathogenesis [50]. FCGs demonstrated the ability to effectively downregulate *nfkb1*, *rela*, *tnf* and *il6*. These anti-inflammatory effects are analogous to those of the flavonoid hesperidin, which has been confirmed to alleviate hepatic inflammation by inhibiting NF-κB, thereby improving MASLD [51].

MASLD progression is linked to increased hepatocyte apoptosis, reflected by a reduced BCL-2/BAX ratio [52,53,54]. Notably, network pharmacology and molecular docking analyses revealed that FCGs exhibited remarkable binding affinities for BCL-2 and BAX, beyond their effects on glycolipid metabolism and inflammation. The qPCR results further corroborate this finding—FCGs significantly enhance the *bcl2*/*bax* ratio, indicating their ability to inhibit the apoptotic pathway. This protective effect may mitigate liver injury and fibrosis, key drivers of MASLD-to-MASH transition.

FCGs ameliorate MASLD through a multi-target mechanism, synergistically addressing insulin resistance, lipid dysregulation, inflammation, and apoptosis. This broad-spectrum activity corresponds well with the multifactorial pathogenesis of MASLD, underscoring therapeutic potential of FCGs. Future studies should explore efficacy of FCGs across MASLD stages and clarify its direct molecular targets.

## 5. Conclusions

In this study, four FCGs were isolated from *Dianthus superbus* L. Based on targets through integrated network pharmacology and molecular docking screening, subsequent zebrafish validation demonstrates that FCGs exert their anti-MASLD effects via a multi-target mechanism, including PPARγ activation, restoration of the INSR-PI3K-AKT pathway, suppression of NF-κB-mediated inflammation and inhibition of hepatocyte apoptosis. These synergistic actions collectively ameliorate key MASLD pathologies, including hepatic lipid accumulation, metabolic dysfunction, tissue damage and enhanced locomotor activity. Our findings demonstrate that FCGs, particularly FCG2 and FCG4, represent promising multi-target candidates for treating MASLD. This natural-agent approach offers a therapeutic strategy against this complex metabolic disorder and warrants further investigation.

## Figures and Tables

**Table 1:** Primers Used for qPCR.

Gene	Forward Primer	Reverse Primer
*β*-*actin*	ACATCAGCATGGCTTCTGCT	GAAGTCCTCTCGGGGAAAGC
*pparg*	TCACTCTCCGCTGATATGGTG	TTGGGTCATTCTGTGTTGGGT
*insr*	TCATCTTCCGCGTCTATGGC	CTGTGGAGGCCGATTTCCTT
*pi3k*	TGTGAAGCACTCAAGCAGTCA	ATCACCGAGGCAGAAAGACG
*akt1*	AAGAGGGGATCACAGACGGA	GTCCTGGTTGTAGAACGGCA
*nfkb1*	AGGCCAAAGACACTGTTCGG	GGAAAGGTTGTGGGGTCCAT
*rela*	CCCGCCATTAGGGTTCACAA	CTCGTGTGGGTGTGGCTTAT
*tnf*	CAAATCACCACACCTTCAGCTTC	CACACCGCCAACCCATTTCA
*il6*	TAAATCCGCATGGACTCGCA	CGGTCCTCTTGGGGTCTTTC
*bcl2*	TTCTAACCGTGGCCGAAGAG	ATCTACCTGGGACGCCATCT
*bax*	TACTTTGCCTGTCGCCTTGT	AGCGAGGAAAACTCCGACTG

**Table 2:** Intermolecular Interactions Between FCGs and Target Proteins.

Proteins	Compound	Binding Energy (kcal/mol)	Binding Residues
PPARγ	FCG1	−5.4	TYR-63
	FCG2	−5.3	MET-22 ILE-37
	FCG3	−5.9	ASN-8 LEU-47 MET-22
	FCG4	−6.0	TYR-63 GLY-62 VAL-60 ASP-58 ASN-46 ARG-47
INSR	FCG1	−7.2	LEU-1002 HIS-1081 SER-1086
	FCG2	−6.7	ALA-1080 ASP-1083
	FCG3	−6.9	ASP-1083 SER-1086 ARG-1089 GLU-1096
	FCG4	−8.1	ASP-1083 LYS-1085
PI3K	FCG1	−9.9	GLN-291 LYS-298 ASP-654 HIS-658 ARG-849
	FCG2	−8.5	GLU-852 ARG-849 GLN-846 GLU-880 ASP-788 ARG-690 ARG-277
	FCG3	−9.5	LYS-298 PRO-866 GLY-868
	FCG4	−10.4	LYS-298 GLN-291 PRO-866 ARG-849 HIS-658 LEU-657 ASP-654
AKT1	FCG1	−7.6	ARG-15
	FCG2	−7.2	GLU-32 ARG-328 ASP-325 ASN-324
	FCG3	−8.6	LYS-276 ASN-279 GLU-85
	FCG4	−7.8	ARG-346 GLY-345 LEU-347 PRO-348
NF-κB1	FCG1	−7.1	ASN-219 GLN-435
	FCG2	−7.2	GLN-439 GLN-435 ASN-257 ASP-261
	FCG3	−8.6	ASP-316 ASN-394 VAL-312 ASN-352 SER-351 TRP-318 ASN-310 ASP-271
	FCG4	−8.2	LYS-299 GLN-435 LYS-431 ASN-257
RELA	FCG1	−7.9	LYS-140 GLU-182 ARG-85
	FCG2	−6.8	TYR-25
	FCG3	−7.8	LYS-122 ARG-132 ASN-42
	FCG4	−7.3	GLU-133 SER-63 ARG-85
TNF	FCG1	−8.0	GLU-110 GLY-108 GLU-107 LYS-103 PRO-102 ASP-104
	FCG2	−8.6	GLU-107 PRO-102 LYS-112
	FCG3	−9.6	GLU-116 CYS-101 PRO-100 SER-99
	FCG4	−8.2	GLU-107 GLY-108 GLY-68 LYS-112
IL6	FCG1	−7.3	ASP-160 LYS-46 ARG-104 GLN-159
	FCG2	−7.0	ASP-140 ASN-144 GLU-93
	FCG3	−6.8	ASN-144 LYS-66
	FCG4	−7.6	LYS-46 GLN-156 ARG-104
BAX	FCG1	−8.0	ASN-104 LEU-47 PRO-130
	FCG2	−6.9	LEU-47 ALA-42 GIN-28 GLU-41
	FCG3	−6.5	ASP-98 ASP-102 MET-99 GLN-52 LYS-57
	FCG4	−7.0	ALA-35 ASN-104
BCL-2	FCG1	−8.3	PRO-163 GLY-162 SER-75
	FCG2	−9.2	ASP-62 ARG-66 ARG-26 ARG-68 LYS-22
	FCG3	−9.1	TYR-67 TYR-161 ARG-66 ARG-26 ARG-68 ARG-65
	FCG4	−9.3	PRO-163 GLU-119 ARG-66 ARG-26

## Data Availability

All study data generated or analyzed during this study are included in the article and Supplementary Information Files.

## Supplementary Materials

The following supporting information can be downloaded at: https://www.mdpi.com/article/10.3390/nu17152456/s1, Figure S1. The optimized analytical (A1) and preparation (A2) chromatogram of the fraction 1 on SunFire® column; the optimized analytical (B1) and preparation (B2) chromatogram of the fraction 2 on Click XION column; and purity assessment of compounds 2″-O-rhamnosyllutonarin, Luteolin 6-C-glucoside-7-O-glucoside, Luteolin 6-C-(6″-O-β-D-glucoside)-glucoside and 6‴-O-rhamnosyllutonarin on SunFire® analytical column (C1, D1, E1 and F1) and Click XION analytical column (C2, D2, E2 and F2); Figure S2. ESI-HRMS of compound a in positive ion mode; Figure S3. ^1^H NMR spectra of compound a; Figure S4. ^13^C NMR spectra of compound a; Figure S5. HSQC spectra of compound a; Figure S6. HMBC spectra of compound a; Figure S7. H-H COSY spectra of compound a; Figure S8. UV spectra of compound a; Figure S9. IR spectra of compound a; Figure S10. ESI-MS of compound b in negative ion mode; Figure S11. ^1^H NMR spectra of compound b; Figure S12. ^13^C NMR spectra of compound b; Figure S13. UV spectra of compound b; Figure S14. IR spectra of compound b; Figure S15. ESI-MS of compound c in negative ion mode; Figure S16. ^1^H NMR spectra of compound c; Figure S17. ^13^C NMR spectra of compound c; Figure S18. UV spectra of compound c; Figure S19. IR spectra of compound c; Figure S20. ESI-MS of compound d in negative ion mode; Figure S21. ^1^H NMR spectra of compound d; Figure S22. ^13^C NMR spectra of compound d; Figure S23. UV spectra of compound d; Figure S24. IR spectra of compound d.

## Abbreviations

The following abbreviations are used in this manuscript:

MASLD	Multidisciplinary Digital Publishing Institute
NAFLD	non-alcoholic fatty liver disease
FCGs	flavone C-glycosides
TG	triglyceride
TC	total cholesterol
LDL-C	low-density lipoprotein cholesterol
HDL-C	high-density lipoprotein cholesterol
PPI	protein-protein interaction
GO	Gene Ontology
KEGG	Kyoto Encyclopedia of Genes and Genomes
