# Supplementary material for: Flavone C-Glycosides from Dianthus superbus L. Attenuate Metabolic Dysfunction-Associated Steatotic Liver Disease (MASLD) via Multi-Pathway Regulations
Source: Nutrients. 2025 Jul 28;17(15):2456. doi: 10.3390/nu17152456 (PMC12348521; doi:10.3390/nu17152456)
Supplement: Supplementary file 1 [file nutrients-17-02456-s001.zip › nutrients-3768387-supplementary.pdf]

Supporting information

Flavone C-Glycosides from Dianthus superbus L. Attenuate Metabolic Dysfunction-Associated Steatotic Liver Disease (MASLD) via Multi-Pathway Regulations

Ming Chu[1], Yingying Tong[2, 3], Lei Zhang[1], Yu Zhang[1, 2], Jun Dang[2, *] and Gang Li[1, *]

[1] Center for Mitochondria and Healthy Aging, College of Life Sciences, Yantai University, Yantai 264005, P. R. China; 18136875242@163.com (M. C.); zhanglei982024@163.com(L.Z.); zhangyu_ytu@126.com(Y.Z.)

[2] Qinghai Provincial Key Laboratory of Tibetan Medicine Research, Key Laboratory of Tibetan Medicine Research, Northwest Institute of Plateau Biology, Chinese Academy of Sciences, Xining 810001, P. R. China; 15371590989@163.com (Y.T.)

[3] Jiangsu Collaborative Innovation Center of Chinese Medicinal Resources Industrialization, Nanjing University of Chinese Medicine, 138 Xianlin Road, Nanjing 210023, P. R. China

* Correspondence: ligang@ytu.edu.cn (G.L.); dangjun@nwipb.cas.cn (J.D.); Tel.: +86-535-6902638 (G.L.); +86-971-6143-282 (J.D.)

Contents

Experimental and results.. 3

Isolation, purity and structural characterization of sample.. 3

The ESI-MS and NMR data for compound a (peak a).. 4

The ESI-MS and NMR data for compound b (peak b).. 5

The ESI-MS and NMR data for compound c (peak c).. 5

The ESI-MS and NMR data for compound d (peak d).. 6

Figures.. 7

Figure S1. The optimized analytical (A1) and preparation (A2) chromatogram of the fraction 1 on SunFire® column; the optimized analytical (B1) and preparation (B2) chromatogram of the fraction 2 on Click XION column; and purity assessment of compounds 2''-O-rhamnosyllutonarin, Luteolin 6-C-glucoside-7-O-glucoside, Luteolin 6-C-(6''-O-β-D-glucoside)-glucoside and 6'''-O-rhamnosyllutonarin on SunFire® analytical column (C1, D1, E1 and F1) and Click XION analytical column (C2, D2, E2 and F2).. 8

Figure S2. ESI-HRMS of compound a in positive ion mode.. 9

Figure S3. ^{1}H NMR spectra of compound a.. 10

Figure S4. ^{13}C NMR spectra of compound a.. 10

Figure S5. HSQC spectra of compound a.. 11

Figure S6. HMBC spectra of compound a.. 11

Figure S7. H-H COSY spectra of compound a.. 12

Figure S8. UV spectra of compound a.. 12

Figure S9. IR spectra of compound a.. 13

Figure S10. ESI-MS of compound b in negative ion mode.. 13

Figure S11. ^{1}H NMR spectra of compound b.. 14

Figure S12. ^{13}C NMR spectra of compound b.. 14

Figure S13. UV spectra of compound b.. 15

Figure S14. IR spectra of compound b.. 15

Figure S15. ESI-MS of compound c in negative ion mode.. 16

Figure S16. ^{1}H NMR spectra of compound c.. 16

Figure S17. ^{13}C NMR spectra of compound c.. 17

Figure S18. UV spectra of compound c.. 17

Figure S19. IR spectra of compound c.. 18

Figure S20. ESI-MS of compound d in negative ion mode. 18
Figure S21. ^{1}H NMR spectra of compound d. 19
Figure S22. ^{13}C NMR spectra of compound d. 19
Figure S23. UV spectra of compound d. 20
Figure S24. IR spectra of compound d. 20

Experimental and results

Isolation, purity and structural characterization of sample

Fraction 1 was analyzed on a SunFire® C18 analytical column (4.6 × 250 mm, 5 μm) using an isocratic elution procedure (mobile phase A: 0.1% *v*/*v* formic acid (FA) in H_2O, mobile phase B: chromatography-grade methanol (MeOH), 0-80 min, 20%-20% B). The chromatographic peak a was obtained (Figure S1A). Fraction 2 was analyzed on a Click XION analytical column (4.6 × 250 mm, 5 μm) using an isocratic elution procedure (mobile phase A: 5% *v*/*v* trifluoroacetic acid (TFA) in H_2O, mobile phase B: chromatography-grade acetonitrile, 0-80 min, 90%-90% B). Three chromatographic peaks (peaks a, b, and c, Figure S1B) were obtained. The analytical conditions for fraction 1 and fraction 2 were linearly amplified at a flow rate of 19.0 mL/min on columns (20 × 250 mm, 5 μm) of the same packing material used for the separation preparation, monitored and collected at 210 nm. The concentrations of compounds a-d were 28.62 mg, 186.68 mg, 2.18 mg, and 49.92 mg, respectively. Click XION analytical columns and SunFire® C18 (4.6 × 250 mm, 5 μm) were used to confirm the purity of compounds a-d. Specific conditions for purity analysis were shown in Figure Note of Figure S1. As shown in Figures S1C-S1F, the purity of the compounds were all over 95%.

After analyzing the obtained nuclear magnetic resonance (HMR, Waters ZQ 2000 (Waters, USA), a Bruker Avance 600 M*Hz* (Bruker, Karlsruhe, Germany)), mass

spectrometry (MS), ultraviolet and visible spectrum (UV, Shimadzu UV2401PC instrument, Kyoto, Japan) and infrared spectroscopy (IR, Bruker, Ettlingen, Germany) data (Figures S2-S24 of Supporting information) and comparing them with the known literature, it was finally determined that the compounds a-d were 2''-O-rhamnosyllutonarin [32, 33], Luteolin 6-C-glucoside-7-O-glucoside [32], Luteolin 6-C-(6''-O-β-D-glucoside)-glucoside [34] and 6'''-O-rhamnosyllutonarin [35], respectively. Figure 1 shows the chemical structures of the isolated compounds.

The ESI-MS and NMR data for compound a (peak a)

Compound a (2''-O-rhamnosyllutonarin, ESI-HRMS *m*/*z* 757.21753, $[M+H]^+$, Figure S2, calc. for $C_{33}H_{40}O_{20}$ *m*/*z* 756.21): ^1H NMR (600 M*Hz*, DMSO-d_6) δ 13.59 (1H, s, 5-OH), 7.44 (1H, dd, *J* = 8.2, 2.0 *Hz*, H-6'), 7.42 (1H, brs, H-2'), 6.90 (1H, d, *J* = 8.2 *Hz*, H-5'), 6.87 (1H, s, H-8), 6.75 (1H, s, H-3), 4.97 (1H, d, *J* = 7.9 *Hz*, H-1''''), 4.95 (1H, brs, H-1'''), 4.71 (1H, dd, *J* = 9.9 *Hz*, H-1''), 4.13 (1H, dd, *J* = 9.9, 8.5 *Hz*, H-2''), 3.61 (1H, brs, H-2'''), 3.58, 3.64 (each 1H, m, H-6''), 3.56, 3.78 (each 1H, m, H-6''''), 3.50 (1H, m, H-5''''), 3.37 (1H, m, H-2''''), 3.36 (1H, m, H-3''), 3.32 (1H, m, H-3''''), 3.28 (1H, m, H-4''), 3.24 (1H, m, H-4''''), 3.18 (1H, m, H-5''), 3.15 (1H, dd, *J* = 9.3 *Hz*, 3.0, H-3'''), 2.88 (1H, dd, *J* = 9.3, 9.3 *Hz*, H-4'''), 2.32 (1H, dq, *J* = 9.3, 6.2 *Hz*, H-5'''), 0.59 (3H, d, *J* = 6.2 *Hz*, H-6'''); ^{13}C NMR (151 M*Hz*, DMSO-d_6) δ 181.9 (C-3), 164.3 (C-1), 162.7 (C-6), 159.3 (C-4), 156.6 (C-8), 150.2 (C-4'), 145.9 (C-3'), 121.1 (C-1'), 119.2 (C-6'), 116.0 (C-5'), 113.4 (C-2'), 110.5 (C-5), 105.0 (C-9), 103.1 (C-2), 102.0 (C-1''''), 100.5 (C-1'''), 94.3 (C-7), 81.1 (C-5''), 78.9 (C-3''), 77.3 (C-5''''), 76.7 (C-2''), 75.7 (C-3''''), 73.8 (C-2''''), 71.6 (C-4''), 71.2 (C-1''), 70.5 (C-2'''), 70.2 (C-3'''), 69.7 (C-4'', 4''''), 68.3 (C-5'''), 60.9 (C-6''''), 60.3 (C-6''), 17.6 (C-6'''). The ^1H NMR, ^{13}C NMR, HSQC, HMBC and H-H COSY signals are shown in Figures S3-S7, and the data

compared with the literature data to identify and designate as 2''-O-rhamnosyllutonarin [1, 2].

The ESI-MS and NMR data for compound b (peak b)

Compound b (Luteolin 6-C-glucoside-7-O-glucoside, ESI-MS *m/z* 609.34, $[M-H]^-$, Figure S10, calc. for $C_{27}H_{30}O_{16}$ *m/z* 610.15): ^1H NMR (600 M*Hz*, DMSO-d_6) δ 13.55 (1H, s, C-5-OH), 7.46 (2H, dd, J = 8.4, 2.3 *Hz*, H-2', 6'), 6.91 (1H, d, J = 8.4 *Hz*, H-5'), 6.85 (1H, s, H-8), 6.76 (1H, s, H-3), 4.99 (1H, d, J = 7.1 *Hz*, H-1'' O-glu), 4.65 (1H, d, J = 9.7 *Hz*, H-1''' C-glu), 4.08~3.01(12H, m, sugar protons); ^{13}C NMR (151 M*Hz*, DMSO-d_6) δ 182.0 (C-4), 164.4 (C-2), 162.5 (C-6), 159.4 (C-10), 156.5 (C-8), 150.0 (C-4'), 145.8 (C-3'), 121.3 (C-1'), 119.2 (C-6'), 116.0 (C-5'), 113.6 (C-2'), 110.6 (C-5), 104.9 (C-9), 103.3 (C-3), 101.2 (C-1''), 93.6 (C-7), 81.0 (C-5''), 78.9 (C-3'''), 77.2 (C-5''), 75.7 (C-3''), 73.8 (C-1'''), 72.9 (C-2''), 72.7 (C-2'''), 70.8 (C-4'''), 69.6 (C-4''), 60.7 (C-6'''), 60.3 (C-6''). The ^1H NMR and ^{13}C NMR signals are shown in Figures S11-S12, and the data agreed with the literature data for Luteolin 6-C-glucoside-7-O-glucoside [1].

The ESI-MS and NMR data for compound c (peak c)

Compound c (Luteolin 6-C-(6''-O-β-D-glucoside)-glucoside, ESI-MS *m/z* 609.34, $[M-H]^-$, Figure S15, calc. for $C_{27}H_{30}O_{16}$ *m/z* 610.15): ^1H NMR (600 M*Hz*, DMSO-d_6) δ 13.61 (1H, s, 5-OH), 7.42 (2H, dd, J = 7.9, 18.9 *Hz*, H-2', 6'), 6.85 (1H, d, J = 7.9 *Hz*, H-5'), 6.81 (1H, brs, H-2), 6.73 (1H, brs, H-8), 4.90 (1H, d, J = 7.4 *Hz*, H-1''), 4.64 (1H, d, J = 9.7 *Hz*, H-1'''), 3.79~3.15(10H, m, sugar protons); ^{13}C NMR (151 M*Hz*, DMSO-d_6) δ 181.9 (C-3), 164.6 (C-1), 162.7 (C-6), 159.5 (C-4), 156.5 (C-8), 151.2 (C-4'), 146.1 (C-3'), 120.2 (C-1'), 119.4 (C-6'), 115.9 (C-5'), 112.9 (C-2'), 110.5 (C-5), 104.8 (C-1'''), 102.8 (C-9), 102.1 (C-2), 93.5 (C-7), 81.1 (C-3''), 78.9 (C-5''), 75.8 (C-3'''), 72.7 (C-5'''), 72.4 (C-1''), 70.9 (C-2'''), 70.8 (C-2''), 69.6 (C-4''), 68.1 (C-4'''), 60.4 (C-6''),

60.3 (C-6'''). The ^{1}H NMR and ^{13}C NMR signals are shown in Figures S16-S17, and the data agreed with the literature data for Luteolin 6-C-(6''-O-β-D-glucoside)-glucoside [3].

The ESI-MS and NMR data for compound d (peak d)

Compound d (6'''-O-rhamnosyllutonarin, ESI-MS *m/z* 755.42, $[M-H]^-$, Figure S20, calc. for $C_{33}H_{40}O_{20}$ *m/z* 756.21): ^{1}H NMR (600 M*Hz*, DMSO-d_6) δ 13.61 (1H, s, 5-OH), 7.45 (1H, dd, *J* = 8.4, 2.0 *Hz*, H-6'), 7.41 (1H, d, H-2'), 6.95 (1H, d, *J* = 8.4 *Hz*, H-5'), 6.77 (1H, s, H-3), 6.75 (1H, s, H-8), 4.99 (1H, d, *J* = 7.1 *Hz*, H-1'''), 4.65 (1H, d, *J* = 9.8 *Hz*, H-1''), 4.63 (1H, brs, H-1''''), 3.94 (1H, dd, *J* = 9.8, 8.7 *Hz*, H-2''), 3.93 (1H, brd, *J* = 12.0 *Hz*, H-6'''), 3.73 (1H, dd, *J* = 3.3, 1.4 *Hz*, H-2''''), 3.68 (1H, m, H-5'''), 3.55 (1H, dd, *J* =9.4, 3.3 *Hz*, H-3''''), 3.54-3.65 (2H, m), 3.51 (1H, m, H-6'''), 3.46 (1H, dq, *J* =9.3, 6.2 *Hz*, H-5''''), 3.34 (1H, m, H-2'''), 3.33 (1H, m, H-3'''), 3.28 (1H, m, H-4''), 3.22 (1H, m, H-3''), 3.20 (1H, m, H-4''''), 3.19 (1H, m, H-4'''), 3.17 (1H, m, H-5''), 1.13 (3H, d, *J* = 6.2 *Hz*, H-6''''); ^{13}C NMR (151 M*Hz*, DMSO-d_6) δ 182.0 (C-4), 164.7 (C-2), 162.5 (C-7), 159.5 (C-5), 156.6 (C-9), 150.0 (C-4'), 145.7 (C-3'), 121.2 (C-1'), 119.4 (C-6'), 116.3 (C-5'), 113.5 (C-2'), 110.5 (C-6), 105.0 (C-10), 103.3 (C-3), 101.3 (C-1'''), 100.6 (C-1''''), 93.5 (C-8), 81.2 (C-5''), 78.9 (C-3''), 75.7 (C-5'''), 75.5 (C-3'''), 73.8 (C-2'''), 72.8 (C-1''), 72.0 (C-4''''), 70.9 (C-3''''), 70.8 (C-2''), 70.4 (C-2''''), 69.6 (C-4'',4'''), 68.4 (C-5''''), 66.2 (C6'''), 60.3 (C-6''), 17.9 (C-6''''). The ^{1}H NMR and ^{13}C NMR signals are shown in Figures S21-S22, and the data compared with the literature data to identify and designate as 6'''-Orhamnosyllutonarin [4].

Figures

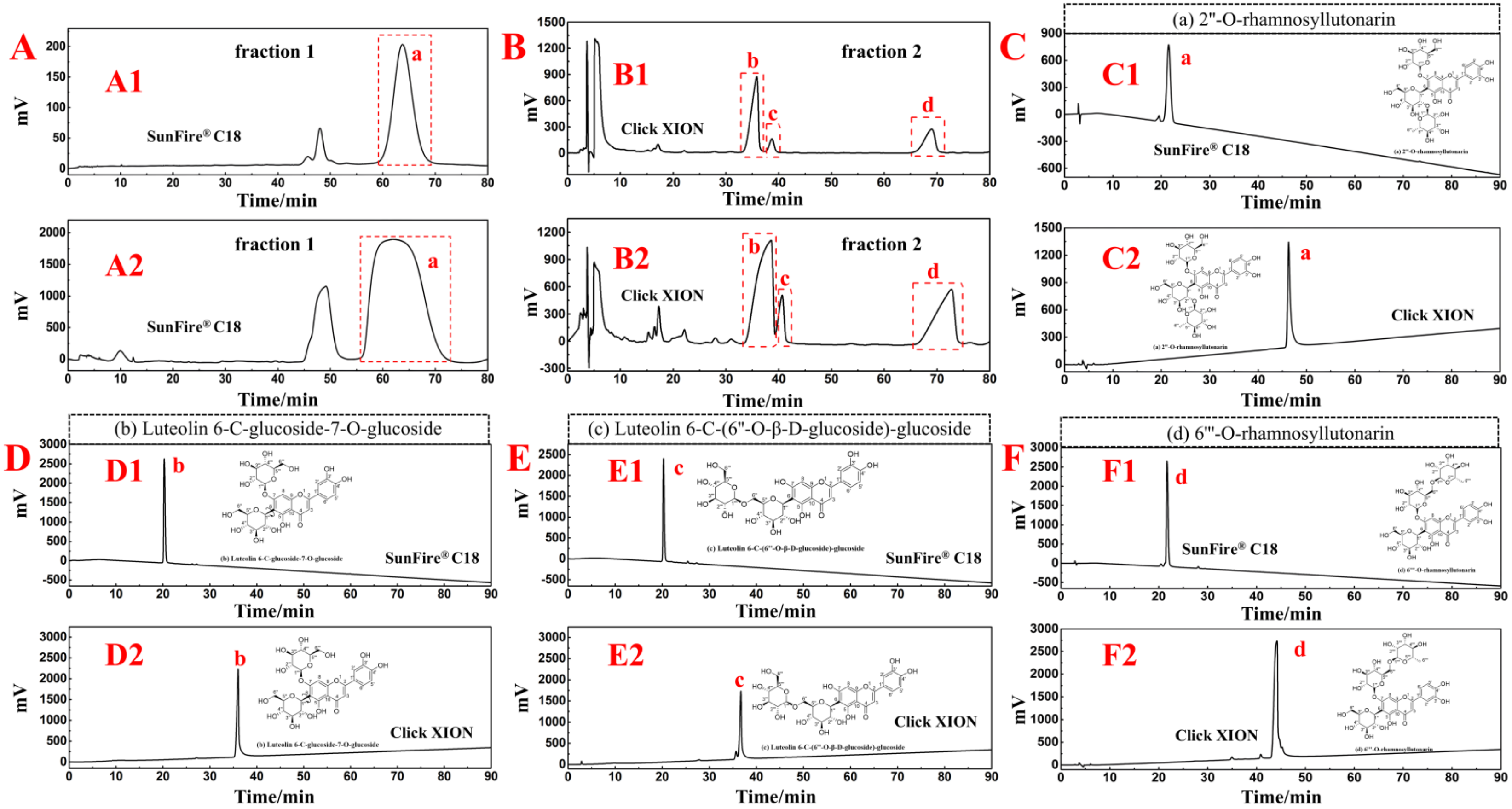

A
A1
fraction 1
SunFire® C18
a
mV
Time/min
A2
B
B1
fraction 2
Click XION
b
c
d
B2
C
(a) 2''-O-rhamnosyllutonarin
C1
C2
D
(b) Luteolin 6-C-glucoside-7-O-glucoside
D1
D2
E
(c) Luteolin 6-C-(6''-O-β-D-glucoside)-glucoside
E1
E2
F
(d) 6'''-O-rhamnosyllutonarin
F1
F2

Figure S1. The optimized analytical (A1) and preparation (A2) chromatogram of the fraction 1 on SunFire® column; the optimized analytical (B1) and preparation (B2) chromatogram of the fraction 2 on Click XION column; and purity assessment of compounds 2''-O-rhamnosyllutonarin, Luteolin 6-C-glucoside-7-O-glucoside, Luteolin 6-C-(6''-O-β-D-glucoside)-glucoside and 6'''-O-rhamnosyllutonarin on SunFire® analytical column (C1, D1, E1 and F1) and Click XION analytical column (C2, D2, E2 and F2).

The chromatogram conditions for fraction 1 were as follows: mobile phase A: 0.1% *v*/*v* FA in chromatographic grade water; B, MeOH; gradient elution, 0-80 min, 20%–20% B; detection wavelength, 210 nm; flow rate, 1.0 mL/min (analysis) and 19.0 mL/min (preparation); column temperature, 35°C. The chromatogram conditions for fraction 2 were as follows: mobile phase A: 5% *v*/*v* TFA in chromatographic grade water; B, ACN; gradient elution, 0-80 min, 90%–90% B; detection wavelength, 210 nm; flow rate, 1.0 mL/min (analysis) and 19.0 mL/min (preparation); column temperature, 35°C. The purity assessment conditions for compounds on SunFire® analytical column (4.6 × 250 mm, 5 µm) were as follows: mobile phase A: 0.1% *v*/*v* FA in chromatographic grade water; B, MeOH; gradient elution, 0-90 min, 20%–80% B; detection wavelength, 210 nm; flow rate, 1.0 mL/min; column temperature, 35°C. The purity assessment conditions for compounds on Click XION analytical column (4.6 × 250 mm, 5 µm) were as follows: mobile phase A: 0.1% *v*/*v* FA in chromatographic grade water; B, ACN; gradient elution, 0-90 min, 90%–60% B; detection wavelength, 210 nm; flow rate, 1.0 mL/min; column temperature, 35°C.

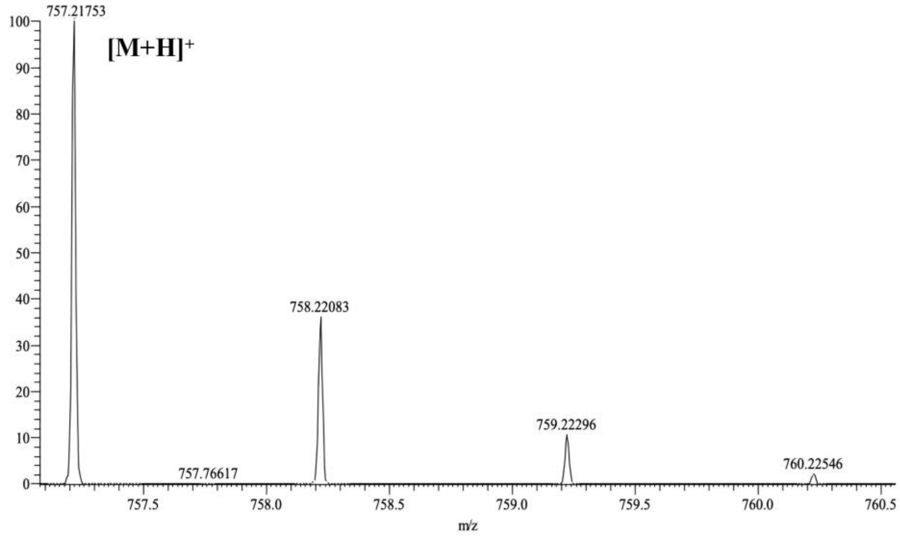

m/z	Theo. Mass	Delta (ppm)	RDB equiv.	Composition	
757.21753	757.21857	-1.37	13.5	C33 H41 O20	M+H

Figure S2. ESI-HRMS of compound a in positive ion mode.

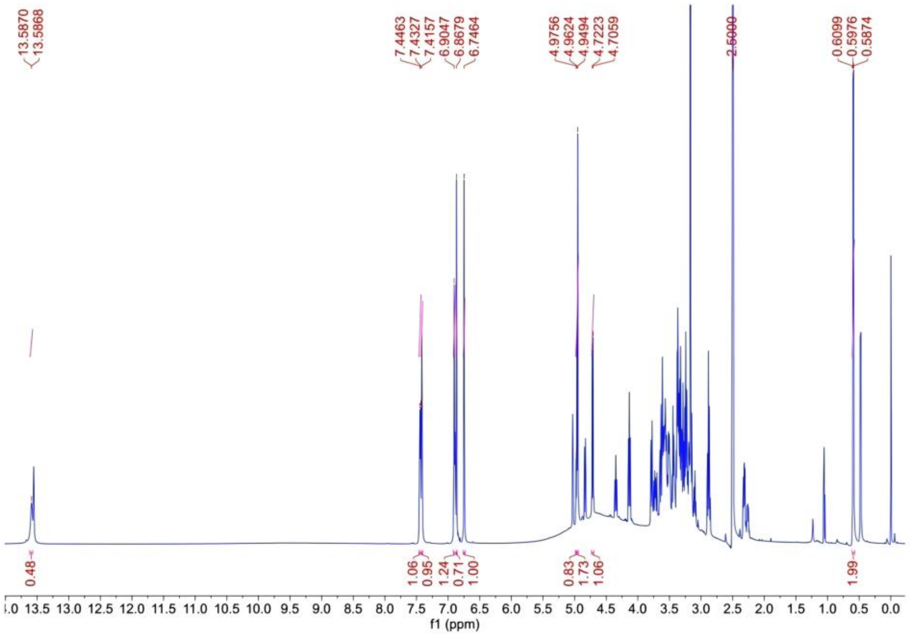

Figure S3. ^{1}H NMR spectra of compound a.

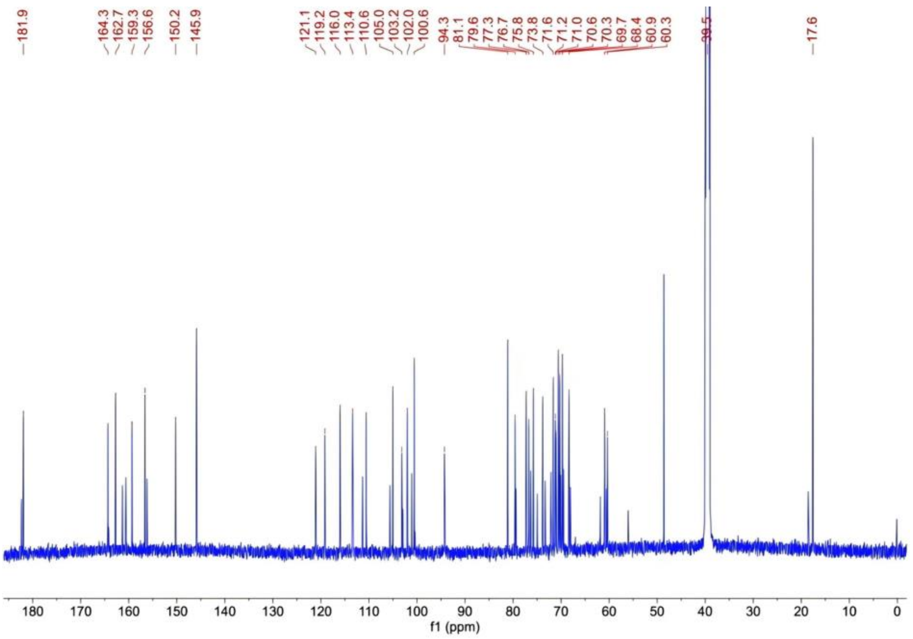

Figure S4. ^{13}C NMR spectra of compound a.

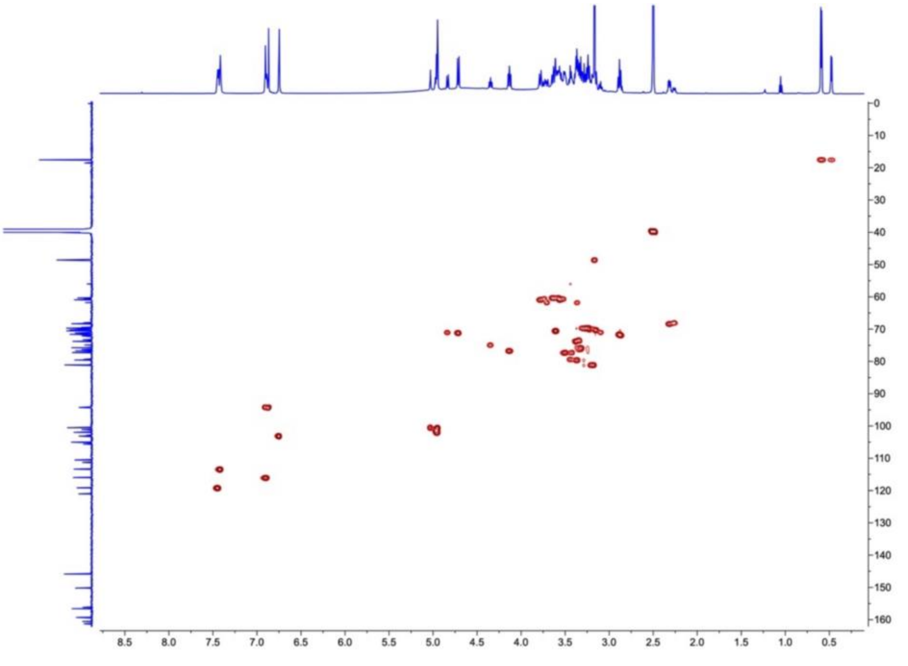

Figure S5. HSQC spectra of compound a.

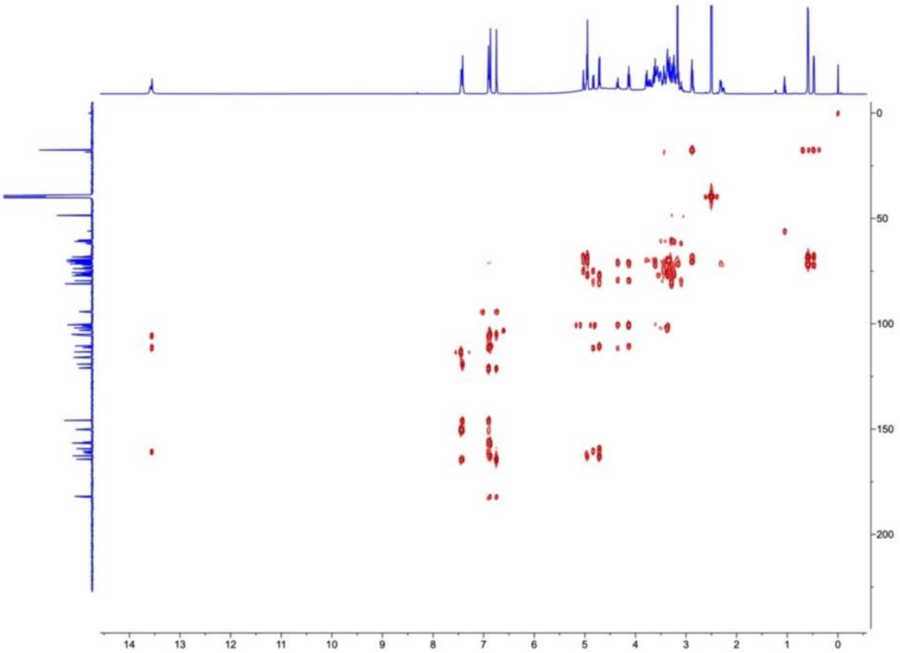

Figure S6. HMBC spectra of compound a.

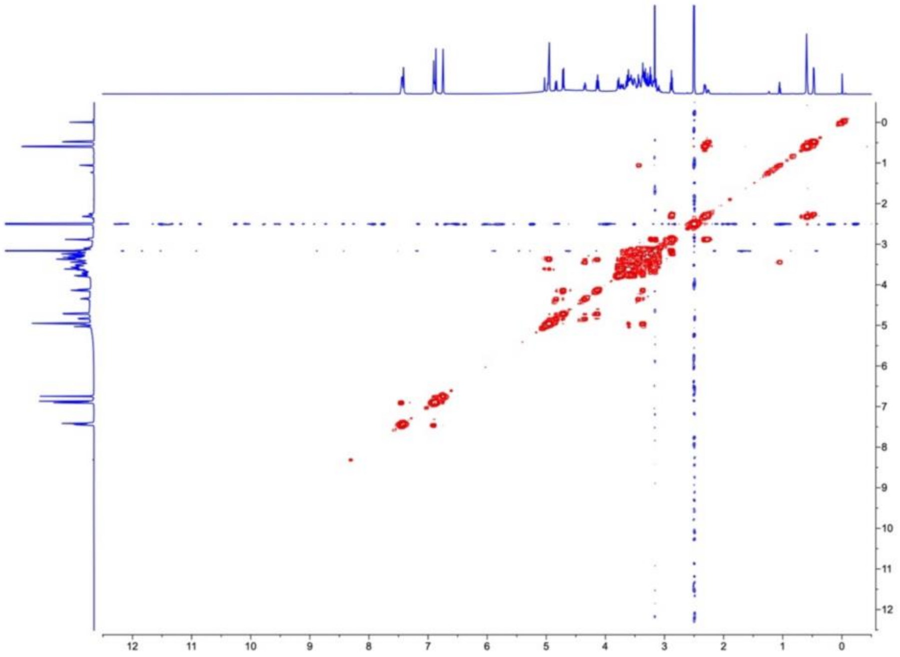

Figure S7. H-H COSY spectra of compound a.

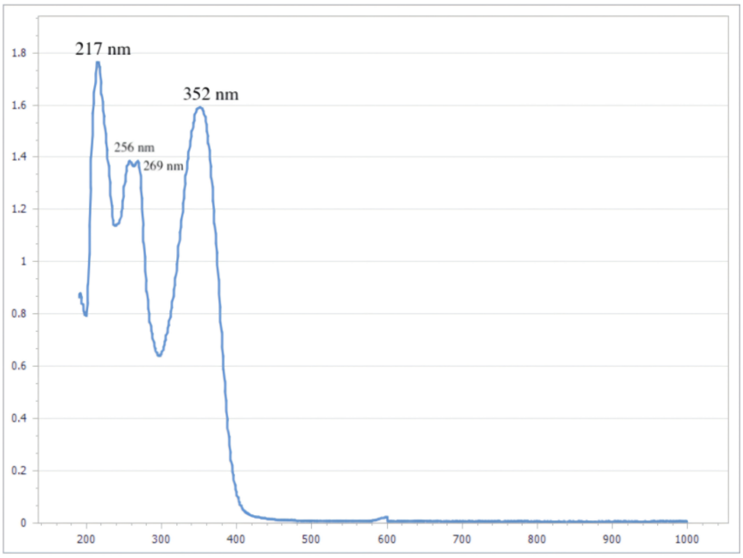

Figure S8. UV spectra of compound a.

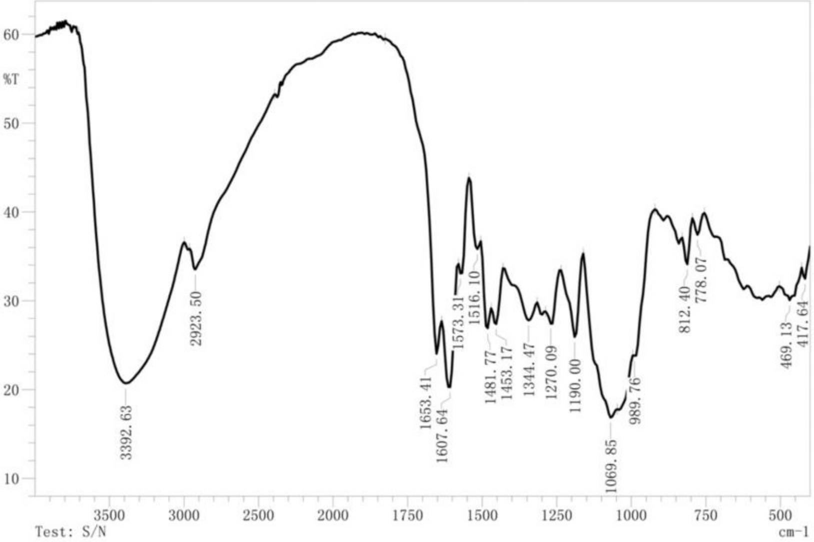

Figure S9. IR spectra of compound a.

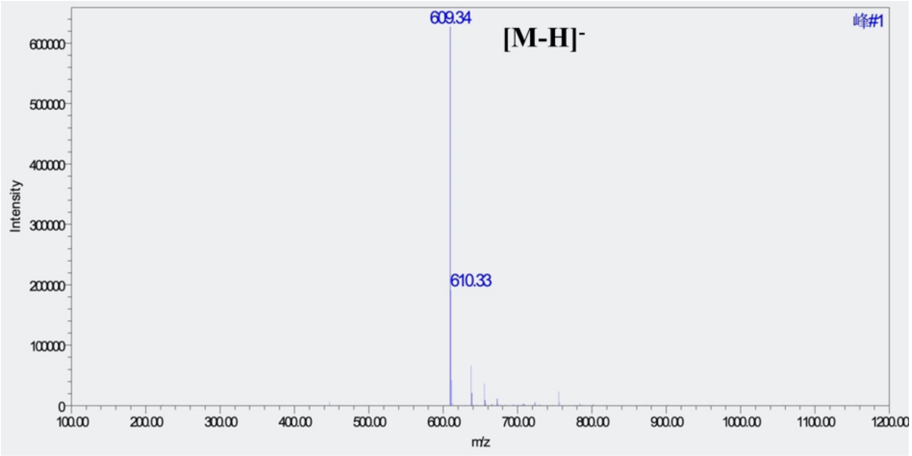

Figure S10. ESI-MS of compound b in negative ion mode.

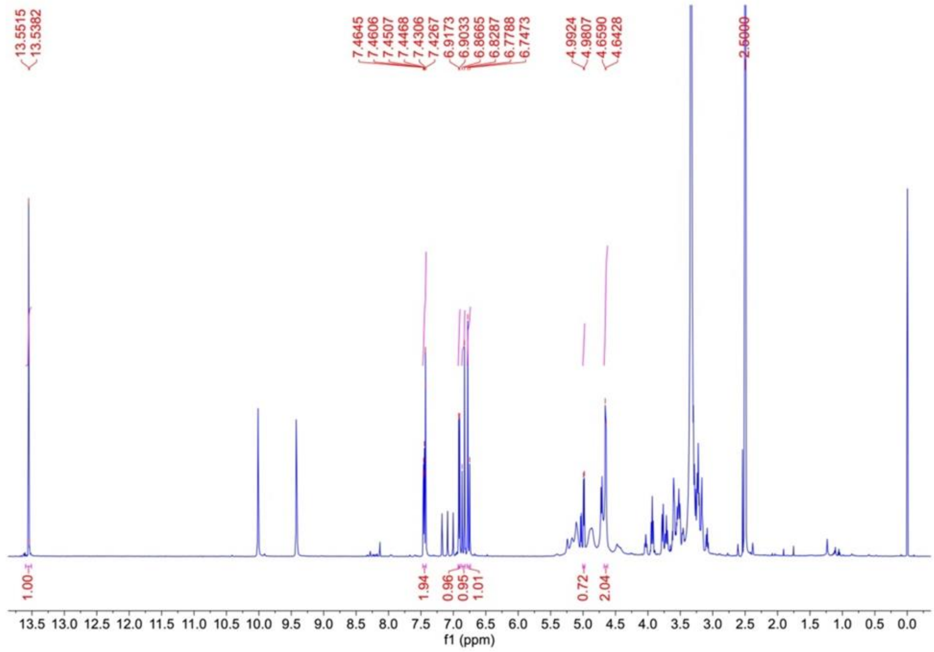

Figure S11. ^{1}H NMR spectra of compound b.

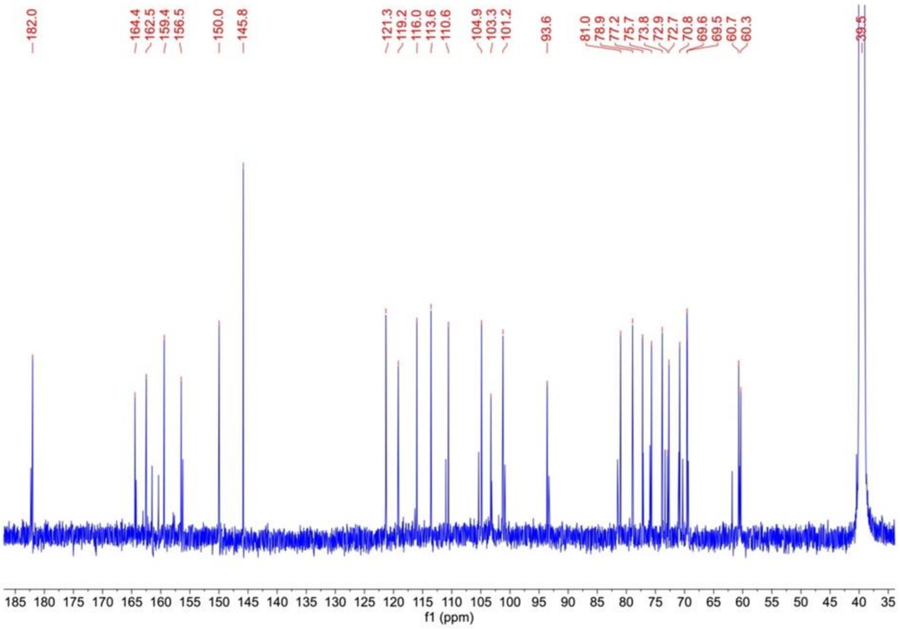

Figure S12. ^{13}C NMR spectra of compound b.

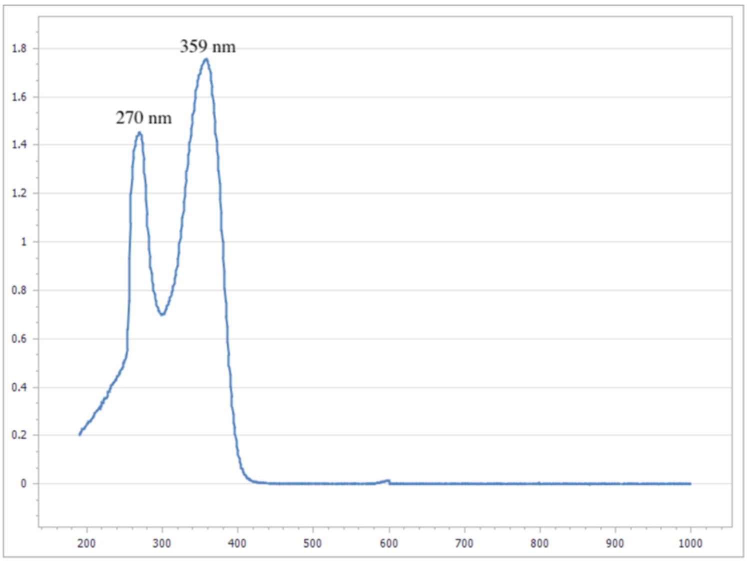

Figure S13. UV spectra of compound b.

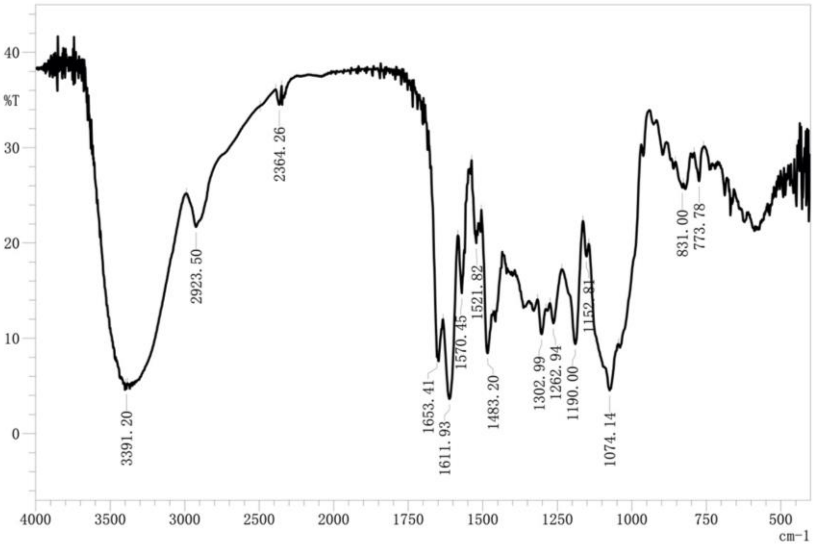

Figure S14. IR spectra of compound b.

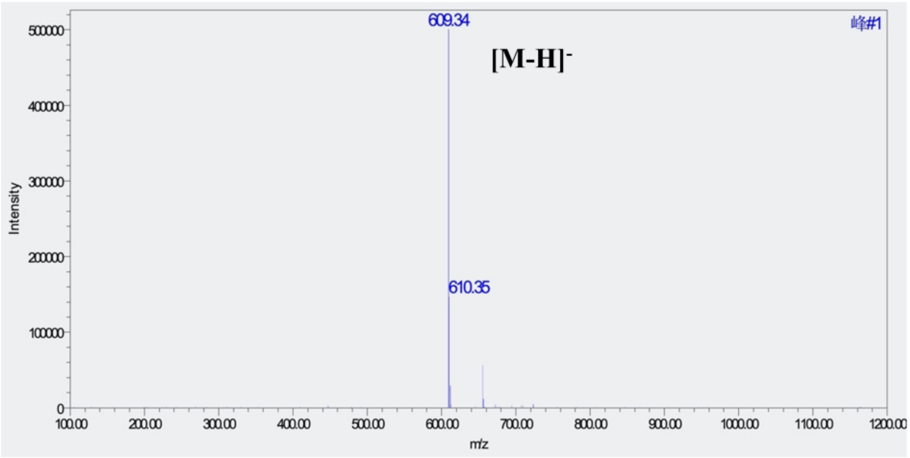

Figure S15. ESI-MS of compound c in negative ion mode.

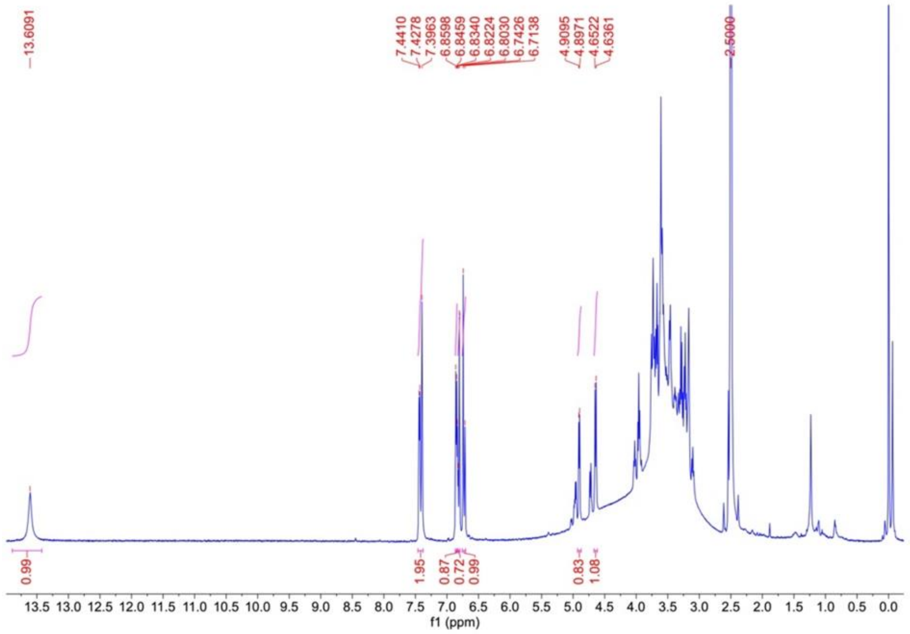

Figure S16. ^{1}H NMR spectra of compound c.

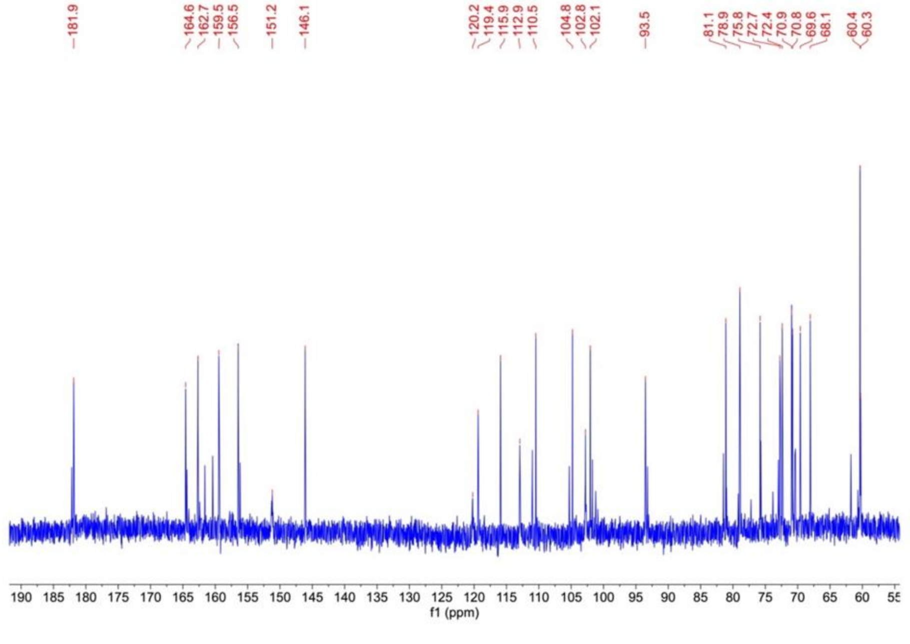

Figure S17. ^{13}C NMR spectra of compound c.

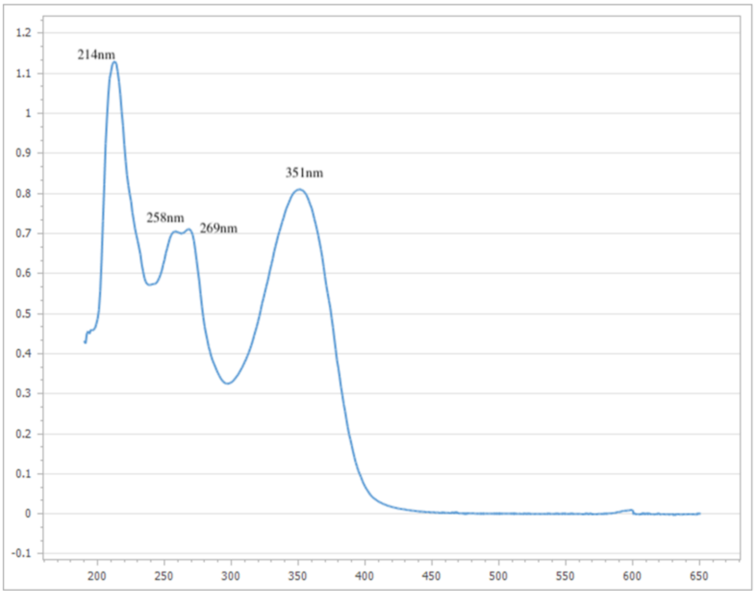

Figure S18. UV spectra of compound c.

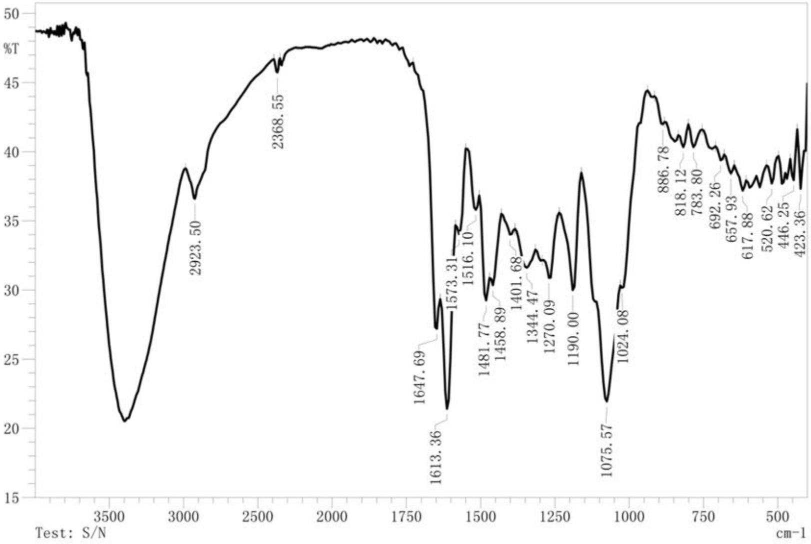

Figure S19. IR spectra of compound c.

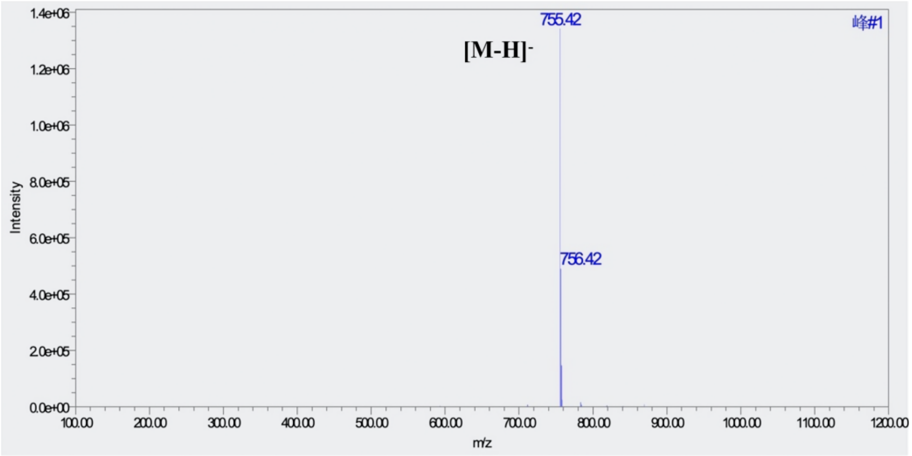

Figure S20. ESI-MS of compound d in negative ion mode.

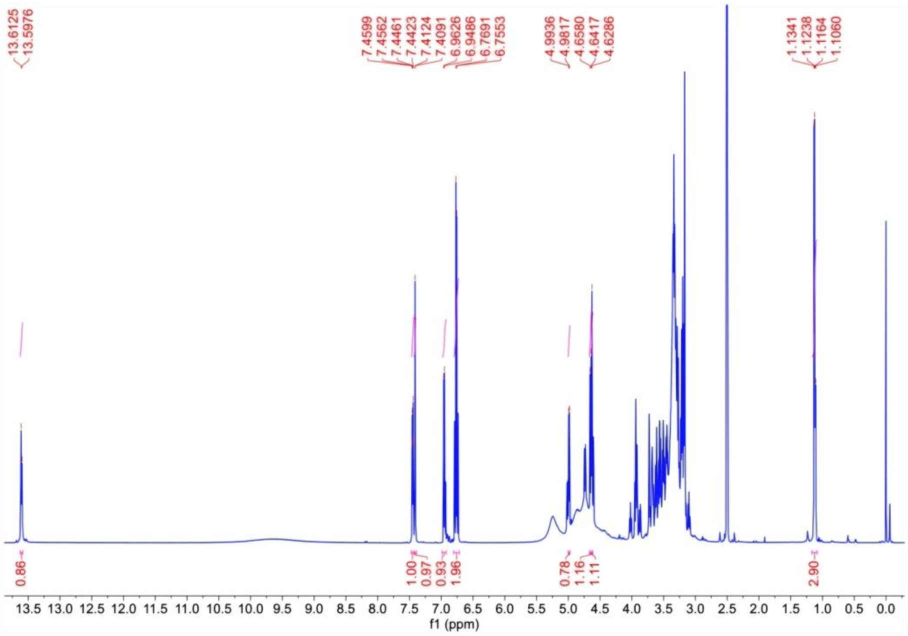

Figure S21. ^{1}H NMR spectra of compound d.

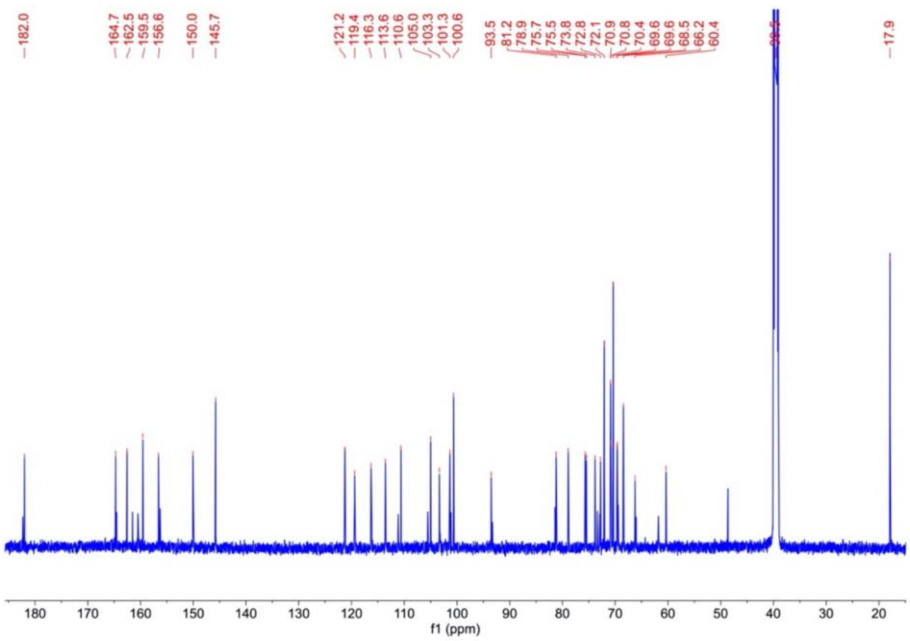

Figure S22. ^{13}C NMR spectra of compound d.

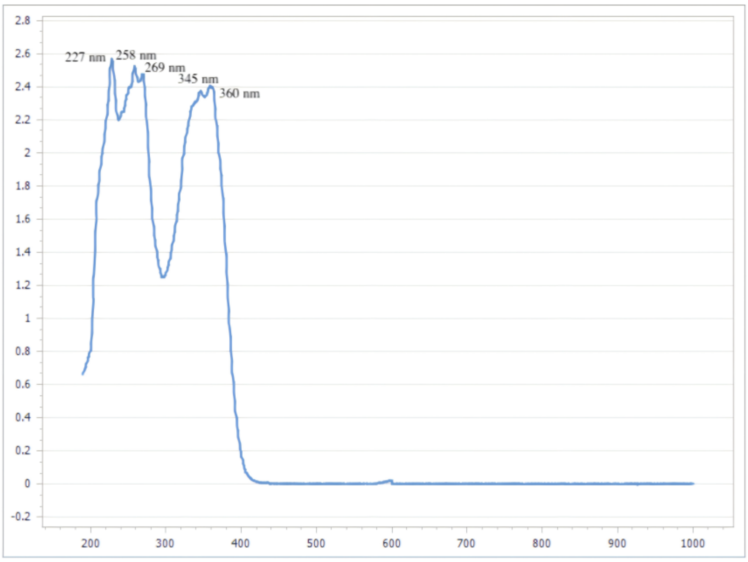

Figure S23. UV spectra of compound d.

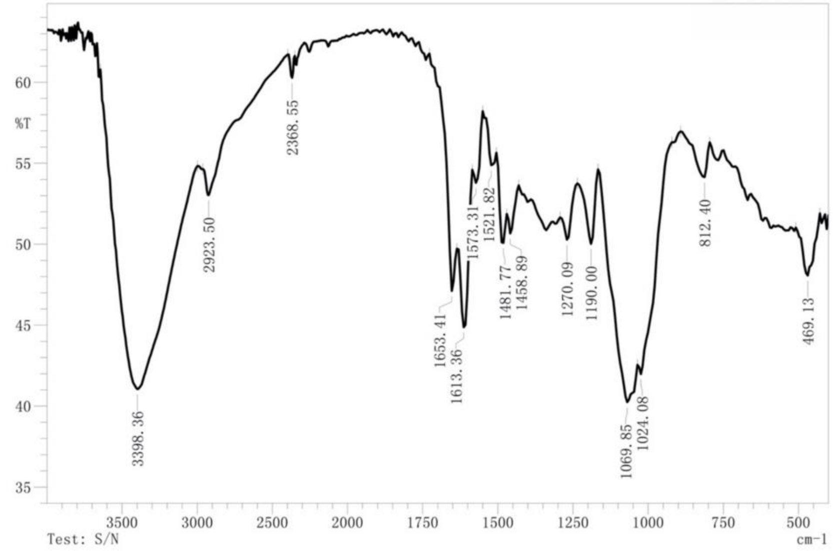

Figure S24. IR spectra of compound d.

References

32. Miroslawa, K.; Wojciech, C. Flavone C-glycosides from Bryonia alba and B. dioica. *Phytochem*. **1995**, *39*(3), 727-729. https://doi.org/10.1016/0031-9422(95)00069-J.

33. Camargo, L.; Férézou, J.P.; Tinoco, L.W.; Kaiser, C.R.; Costa, S.S. Flavonoids from *Mimosa xanthocentra* (Leguminosae: Mimosoideae) and molecular modeling studies for isovitexin-2″-O-α-L-rhamnopyranoside rotamers. *Phytochem Lett*. **2012**, *5*(3), 427–431. https://doi.org/10.1016/j.phytol.2012.03.015.

34. Kuo, S.H.; Yen, M.H.; Chung, M.I.; Lin, C.N. A flavone C-glycoside and an aromatic glucoside from *Gentiana* species. *Phytochem*. **1996**, *41*(1), 309–312. https://doi.org/10.1016/0031-9422(95)00528-5.

35. Obmann, A.; Werner, I.; Presser, A.; Zehl, M.; Swoboda, Z.; Purevsuren, S.; Narantuya, S.; Kletter, C.; Glasl, S. Flavonoid C- and O-glycosides from the Mongolian medicinal plant *Dianthus versicolor* Fisch. *Carbohydr Res*. **2011**, *346*(13), 1868–1875. https://doi.org/10.1016/j.carres.2011.04.031.
